# Cultivable *Winogradskyella* species are genomically distinct from the sympatric abundant candidate species

**DOI:** 10.1038/s43705-021-00052-w

**Published:** 2021-09-29

**Authors:** Carlota Alejandre-Colomo, Ben Francis, Tomeu Viver, Jens Harder, Bernhard M. Fuchs, Ramon Rossello-Mora, Rudolf Amann

**Affiliations:** 1 Marine Microbiology Group, Department of Animal and Microbial Biodiversity, Mediterranean Institute for Advanced Studies (IMEDEA, UIB-CSIC), Miquel Marques 21, 07190 Esporles, Spain; 2 Department of Molecular Ecology, Max Planck Institute for Marine Microbiology, Celsiusstrasse 1, D-28359 Bremen, Germany

**Keywords:** Microbial ecology, Metagenomics

## Abstract

*Winogradskyella* is a genus within the phylum *Bacteroidetes* with a clear marine origin. Most members of this genus have been found associated with marine animals and algae, but also with inorganic surfaces such as sand. In this study, we analyzed genomes of eleven species recently isolated from surface seawater samples from the North Sea during a single spring algae bloom. Corresponding metagenomes yielded a single *Candidatus* species for this genus. All species in culture, with the exception of *W. ursingii*, affiliated with a *Winogradskyella* lineage characterized by large genomes (~4.3 ± 0.4 Mb), with high complexity in their carbohydrate and protein degradation genes. Specifically, the polysaccharide utilization loci (PULs) were diverse within each individual strain, indicating large substrate versatility. Although present in the North Sea, the abundances of these strains were at, or below, the detection limit of the metagenomes. In contrast, the single species, classified as *Candidatus* W. atlantica, to which all North Sea MAGs belonged, affiliated with a lineage in which the cultivated representatives showed small genomes of ~3.0–3.5 Mb, with the MAGs having ~2.3 Mb. In *Ca*. W. atlantica, genome streamlining has apparently resulted in the loss of biosynthesis pathways for several amino acids including arginine, methionine, leucine and valine, and the PUL loci were reduced to a single one for utilizing laminarin. This as-yet uncultivated species seems to capitalize on sporadically abundant substrates that are released by algae blooms, mainly laminarin. We also suggest that this streamlined genome might be responsible for the lack of growth on plates for this *Candidatus* species, in contrast to growth of the less abundant but coexisting members of the genus.

## Introduction

Marine microbiologists commonly find that environmentally abundant taxa are challenging to cultivate (e.g. [1–6]), while many rare taxa can be cultivated quite readily on plates and in liquid media. Culture-independent technologies greatly broaden our understanding of the ecological dynamics of microbial populations. However, the discrepancy in cultivation success has resulted in disjunct sets of genomic and physiological data for cultivated versus environmental taxa (see, for example, the elucidation of nitrogen fixation in cultivated and uncultivated cyanobacteria [7] and the identification of diazotrophy genes in other phyla [3]). There is thus an incentive to better understand how to cultivate ecologically relevant taxa in order to be able to test hypotheses generated from the analysis of genomic and metagenomic data.

Recently, we described eight novel species of the *Bacteroidetes* genus *Winogradskyella*, a widespread marine genus typically isolated from macroalgae [8–10] and marine invertebrates [11–13]. Our strains, however, were isolated from North Sea surface water during the 2016 spring phytoplankton bloom [14]. In the study, we also described the novel candidate species *Candidatus* W. atlantica, based on 14 metagenome-assembled genomes (MAGs) recovered from sampling campaigns across multiple spring blooms [14]. The high-throughput cultivation effort on plates that yielded those novel species, part of a total of 41 strains from ten *Winogradskyella* species, did not provide a single representative of the candidate taxon among the ~5000 isolates [15], and none of the cultivated species was represented by a MAG.

Regardless of their natural habitat, a prominent feature of *Bacteroidetes* is the presence of polysaccharide utilization loci (PULs) [16, 17]. These are specialized genetic loci encoding poly- and oligosaccharide transport with degradation function via co-located carbohydrate active enzymes (CAZymes) [18]. PUL content is one feature where divergence appears between genomes of cultivated and uncultivated taxa. Genomes of isolated *Bacteroidetes* species from the North Sea, for example, have been found to possess on average seven PULs, with a range beyond 20 in some cases [19]. Meanwhile in MAGs of spring bloom-associated North Sea *Bacteroidetes*, the number of PULs has been found to be on average closer to three or four, and with a smaller overall range [20] in spite of the high concentrations of algal derived polysaccharide [21–24]. The so-called ‘selfish’ [25] consumption of polysaccharide encoded by PULs is expected to be important in constraining the ecological niches of the different *Winogradskyella* species studied here.

Members of the *Bacteroidetes* also encode a large number of peptidases which avail them of a high proteolytic potential [16, 19, 26–28]. Non-marine *Bacteroidetes* have been shown to have similar numbers of CAZymes and peptidases [29], while planktonic *Bacteroidetes* often show higher ratios of peptidases to degradative CAZymes [28]. Contrastingly, CAZyme-rich species with lower peptidase numbers include known algae-associated species such as *Zobellia galactanivorans* Dsij^T^ *Formosa agariphila* KMM 3901^T^ [28], reflecting their polysaccharide rich natural habitats.

Following algae blooms, soluble cytosolic polysaccharides with a limited structural diversity, i.e., laminarin, become a dominant substrate. This is reflected in the observed differences in inferred bacterial genomic polysaccharide degradation capacities [19, 20]. This alone should not be enough to directly affect cultivability on plates, but the species most readily identified in metagenomes must have other features that prevent them from growing under the laboratory conditions. The most obvious point of difference between strains that can be isolated and those that cannot is genome size, with MAGs of environmentally abundant *Bacteroidetes* being smaller on average than those of isolated strains [19, 20]. Narrowing of carbohydrate substrate preferences has been suggested as one route to smaller genome size in the gammaproteobacterial genus *Idiomarina* [30]. While not as small as ubiquitous marine oligotrophs such as the SAR11 [31] or OM43 [32] clades, genome streamlining in bloom-associated *Bacteroidetes* could support the efficient growth needed to reach the large populations observed during phytoplankton blooms. Streamlining to maintain only essential genes for a specific environment implies various consequences relevant for cultivation success, with the most significant being reliance on other organisms for essential vitamins or amino acids due to the loss of these biosynthetic capabilities [33, 34]. Mostly this theory has been applied to explain the ubiquity of purely oligotrophic organisms, however, rather than to organisms that combine this with seasonal copiotrophy [35].

In the present study, we leverage the outcome of a large-scale isolation effort during the North Sea spring bloom of 2016 [15] to investigate genomic differences between readily isolated *Winogradskyella* species and the uncultivated candidate species abundant in the water column at that time.

## Materials and methods

### Selection, sequencing, and assembly of *Winogradskyella* strains genomes

*Winogradskyella* strains were isolated from surface water in a high-throughput culturing approach performed during the spring bloom of 2016 [15] at the ‘Kabeltonne’ station of the Helgoland Roads (54° 11′ 17.88″ N, 7° 54′ 0″ E). For biomass production, all strains were grown on Marine Agar according to ZoBell (Condalab, Madrid, Spain) at 12 °C. DNA extraction was performed as previously described [36]. Species were initially identified as Operational Phylogenetic Units (OPUs) [15, 37], and 41 strains were characterized with RAPD fingerprints with the primer RAPD1 as previously reported [38]. Strains with different RAPD patterns were chosen to avoid strain clonality within species. Fifteen strains were sequenced with Illumina HiSeq3000 (Illumina, San Diego, CA, USA), and, in addition, strains Z354, Z963, SW133, HL6110, HL857 and Z215 were also sequenced with Pacific Biosciences PacBio RS. Illumina reads were trimmed with PHRED score quality threshold of 20 using SolexaQA v3.1.4 [39] and trimmed reads were assembled using SPADES 3.10 [40]. Hybrid assembly for genomes sequenced using Illumina and PacBio platforms (Supplementary Table S1) was performed using the --pacbio option of SPADES 3.10.

Genome completeness was determined using the MiGA tool [41]. Translated sequences of single-copy house-keeping genes [42] were used for phylogenetic purposes. After aligning the genes using MUSCLE v3.8.31 [43], the genes were concatenated and used to build phylogenetic trees using the RAxML and Neighbor-joining algorithms implemented in ARB v6.0.6 [44].

The average nucleotide identity (ANI) and the average amino-acid identity (AAI) between all genomes was determined according to Konstantinidis and Tiedje [45] using the enve-omics webserver (http://enve-omics.gatech.edu/) [46]. AAI values were calculated using core-genome genes after comparing all the genomes from the genus *Winogradskyella* and adding two *Psychroserpens* genomes as an outgroup. ANI and AAI values were represented in heatmaps using the Gplots package from R Studio v1.1.447 [47].

Eighteen *Winogradskyella* genomes were available from NCBI and GTDB databases and were included in the genome comparisons of this study (genome accession code has been included in Supplementary Table S1).

### Metagenomic sequencing, assembly and MAG recovery

MAGs from Helgoland belonging to *Ca*. W. atlantica are part of BioProject PRJEB28156, and have been published and named previously [14, 20]. Abundance estimation of all *Winogradskyella* genomes and MAGs was performed as previously described by Orellana [48].

Four additional *Winogradskyella* MAGs available in the GTDB database [49], three from the Tara Oceans Consortium [50] and one from the University of Queensland [51] and a further Tara Oceans MAG [52] from the MiGA database [41] were also included in this study (Supplementary Table S1).

### Phylogenetic tree reconstruction based on 16S rRNA genes

16S rRNA sequences from the genomes were extracted using barrnap v0.9 (see https://github.com/tseemann/barrnap) [53]. All available *Winogradskyella* 16S rRNA sequences from described species and sequences from the genomes were imported to the reference database LTP_SSU_132 and aligned using SINA v.1.3.1 tool (SILVA Incremental Alignment [54]) implemented in the ARB software package v6.0.6 [44]. Final alignments were manually improved following the reference alignment in ARB-editor. Complete sequences were used to reconstruct *de novo* trees using Neighbor-Joining [55] and RAxML [56], with a conservational filter for bacteria (i.e., allowing all homologous positions of the members of the domain *Bacteria* in the universal alignment implemented in the ARB program package [44]), using Jukes-Cantor and GTRGAMMA corrections, respectively. The partial sequence from MAGs 20110512_Bin_69_1 and MAG_SP247 were added to the reference tree using the built in ARB parsimony tool.

### Core and pan-genome analysis

To identify core- and pan-genomes, predicted protein sequences of genomes and MAGs from the North Sea and available genomes from other locations, were compared pairwise, in an ‘all-versus-all’ manner, using BLAST v2.2.31+ [57]. Proteins were identified as homologous using rbm.rb script implemented in the Enve-omics collection [46] if they shared 50% sequence similarity over 50% or more of the query sequence length. Orthologous groups (OGs) in reciprocal best matches were identified using ogs.mcl.rb script [46]. Those genes present in two or more genomes but not in all genomes were defined as variable genes. The presence/absence of variable genes was used to cluster the genomes with the Euclidean distance using the Ggplot2 package from R Studio v1.1.447 [58]. All proteins shared between all sequenced genomes (core-genes) were aligned using MUSCLE v3.8.31 [43]. Aligned genes were concatenated using Aln.cat.rb script [46]. Orthologous genes, concatenated and aligned, were used to build phylogenetic trees in ARB using Neighbor-Joining [55] and RAxML [56].

### Gene and PUL annotation

Gene prediction and annotation was performed as previously described [19]. Shortly, initial annotations of the genomes were performed using GenDB v2.4 [59] and KEGG [60]. The DOE-JGI MGAP was used to annotate SusC-like proteins with the TIGRFAM model TIGR04056, and SusD-like proteins with the Pfam models PF12741, PF12771, and PF14322. Annotations of CAZymes were done using the dbCAN v6 database [61] and BLASTp searches [57] against the CAZy database v07312018 [18]. Only genes positive against both dbCAN and the CAZY database were considered reliably annotated as CAZymes. SulfAtlas v1.0 [62] was used to annotate sulfatases. The MEROPS 9.13 database [63] was used to annotate peptidases with a default threshold *E* ≤ 10^−4^. PULs were detected based on the presence of CAZyme clusters using a 7 gene sliding window where any CAZyme apart from glycosyl transferases, and any TonB-dependent transporter (including *susC*-like genes), sulfatase, or *susD*-like gene would be considered part of a PUL if located within seven genes up or downstream of any other such gene. The window size of seven was chosen as a balance between the strictness of PULDB’s five gene window [64], and the inclusiveness of the ten gene window used in ref. [20].

### V4 16S rRNA gene amplicon sequencing

Over the years 2010–2012 the 16S rRNA genes of *Winogradskyella* from seawater fraction 0.2–3 μm (small fraction) and 3–10 μm (larger fraction) were sampled and analyzed as described in Chafee [65]. The V4 region of the 16S rRNA gene was amplified using primers 515F (5′-GTGCCAGCMGCCGCGGTAA-3′) and 806R (5′-GGACTACHVGGGTWTCTAAT-3′) and sequenced by Illumina MiSeq 2 × 250 bp chemistry as described previously [66]. Amplicon sequences were then clustered using minimum entropy decomposition (MED) [67], as described in ref. [65].

## Results

### High-quality genome sequences of the *Winogradskyella* strains isolated from the North Sea

**Fig. 1:**
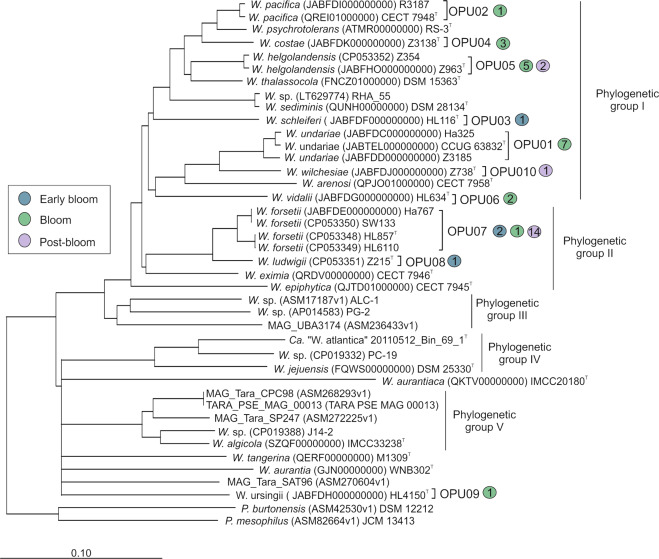
Consensus phylogenetic reconstruction based on Neighbor-Joining algorithm (Supplementary Fig. S6) using the core genome. *Candidatus* “W. atlantica” Bin_69_1^T^ is representative for all fifteen North Sea MAGs. Branches that were not supported by all tree reconstruction algorithms are shown as multifurcations (Supplementary Figs. S4–S6). Colored circles represent the number of strains found in each phase of the sampling.

Our high-throughput cultivation in 2016 [15] yielded the isolation of 41 strains of the genus *Winogradskyella*. The 16 S rRNA gene sequence affiliated them with ten different species, eight of which we have recently classified as new taxa [14]. The phylogeny of the core genome supported this classification (Fig. 1). After dereplication using RAPD profiles (Supplementary Fig. S1), complete genomes of 15n strains were sequenced (Table 1 and Supplementary Table S1), with at least one non-clonal representative from each clade. For the three species with most isolates, namely *W. undariae* (OPU01), *Winogradskyella* *helgolandensis* (OPU05) and *Winogradskyella* *forsetii* (OPU07), three, two and four strains respectively were sequenced, choosing those isolated from different sampling times (Supplementary Fig. S1). In all cases, the sequencing coverage was over 140×. For six of the fifteen strains, we obtained closed genomes (Table 1 and Supplementary Table S1). Each of these genomes contained a single chromosome without plasmids.

**Table 1:**
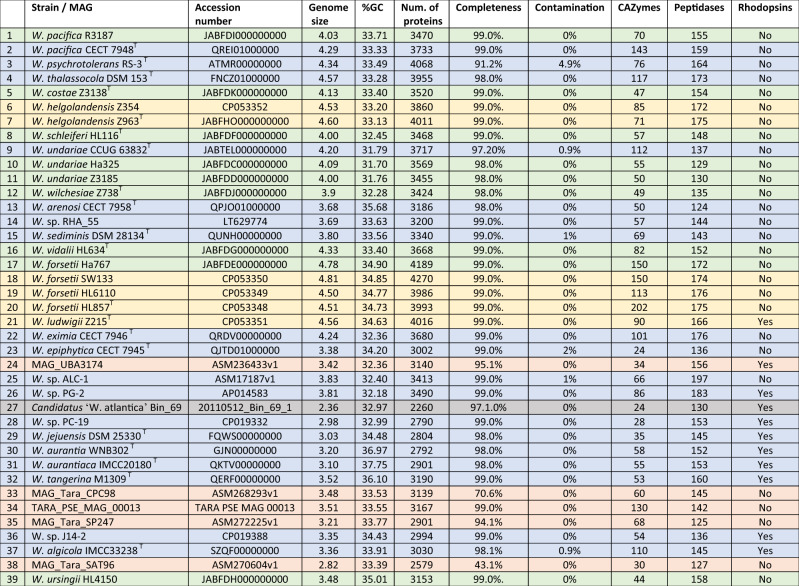
Main features of genomes included in the study.

Candidatus ‘W. atlancia’ 20110512_Bin_69_1 is representative for 14 North Sea MAGs. Strains shaded in green are genomes sequenced by Illumina HiSeq3000, strains shaded in yellow are hybrid assembly for genomes sequenced using Illumina and PacBio platforms, strains shaded in blue are available *Winogradskyella* genomes in public databases, the genome shaded in gray is the representative North Sea MAG and genomes shaded in orange are *Winogradskyella* MAGs available in public databases.

In total, 33 complete genomes were analyzed. Fifteen of these were from Helgoland and 18 genomes obtained from other locations and available in public repositories (Table 1 and Supplementary Table S1). Among them, *Winogradskyella* sp. PC-19 and *W. forsetii* SW133 represented the smallest and largest genomes, of 2.98 Mb and 4.81 Mb, respectively, as well as the lowest and highest number of predicted proteins with 2760 and 4209 (Supplementary Table S1). In addition, *W. undariae* Ha325 showed the lowest G + C mol% content with 31.70%, and *Winogradskyella* *aurantiaca* IMCC20180^T^ the highest with 37.75%. The collection of genomes represented a total of 25 distinct species. Within species, reciprocal ANI values were greater than 95% [68], while between species these were lower than 91.54% (Supplementary Table S2A, Supplementary Fig. S2). The four isolates of *W. forsetii* (OPU07) were considered as two different genotypes because they always formed two independent phylogenetic clades, and the ANI values ranged between 96.69% and 96.70% among the different genotypes and between 99.98% and 100% within a genotype (Supplementary Table S2A). These organisms also showed differences in their putative degradative targets (see further results). Thus, strains SW133 and Ha767 were considered genotype I and strains HL857^T^ and HL6110 genotype II.

### MAGs retrieved from the North Sea samples and identified as members of the genus *Winogradskyella*

From the extensive metagenomic data available from Helgoland from the years 2010–2016, 14 *Winogradskyella* MAGs were recovered, all of them belonging to the same species *Candidatus* W. atlantica with ANI and AAI values >96.5% (Supplementary Table S2B, Supplementary Figs. S 2 and 3). All but the three MAGs recovered from 2011 (Bin 40_1, Bin_70_1 and Bin_46_1) showed within-group ANI and AAI values >99%, indicating a strong temporal homogeneity. Five additional MAGs, representing as yet unclassified taxa, and available in public databases (GTDB [49] and MIGA [41]), came from four additional species based on their ANI values (Supplementary Table S2A). All but six MAGs had completeness values >90% (Supplementary Table S1). Of those more than 90% complete, lengths ranged from 2.20 Mb (*Ca*. W. atlantica Bin_72_1, 97.1% completeness) to 3.51 Mb (TARA_PSE_MAG_00013, 99% completeness), and both also showed the lowest and highest number of predicted proteins of 2,126 and 3,170 respectively. The lowest G + C mol% was for MAG_UBA3174 with 32.36% and the highest was in MAG_Tara_SP247 with 33.77%.

### Genome and 16S rRNA gene phylogenies

Phylogenetic reconstructions showed consistent topologies regardless of the sequences used to reconstruct them. Specifically, (i) the 16S rRNA genes (Supplementary Fig. S4), (ii) the concatenated sequences of twenty-one conserved single-copy orthologous genes (essential genes; Supplementary Fig. S5), (iii) the concatenation of the core genome (Fig. 1; Supplementary Fig. S6), and (iv) AAI/ANI distance dendrograms (Supplementary Figs. S2–S3) showed that the *Winogradskyella* genus could be divided in two main phylogenetic lineages: a major clade in which the type species of the genus (*W. thalassocola*) lies, and which we thus refer as the “thalassocola” branch, and a smaller clade in which the majority of environmental MAGs affiliated, henceforth the “atlantica” branch (taking *Ca*. W. atlantica as the reference for the clade). All North Sea isolates except *Winogradskyella* *ursingii* HL4150^T^ (OPU09) affiliated with the “thalassocola” branch. This major branch was formed by three individual lineages designated as phylogenetic groups I (PGI), II (PGII) and III (PGIII). On the other hand, all MAGs with the exception of MAG UBA_3174, affiliated with the “atlantica” branch formed by two individual clades. The clade containing all North Sea MAGs together with strains *Winogradskyella* sp. PC-19 and *Winogradskyella* *jejuensis* DSM25330^T^ was designated as phylogenetic group IV (PGIV), while the branch formed by MAGs from the Tara Oceans Expedition and the strains *Winogradskyella* sp. J14-2 and *Winogradskyella* *algicola* IMCC33238^T^ were designated as phylogenetic group V (PGV). *Winogradskyella* *epiphytica, Winogradskyella aurantiaca* IMCC20180^T^, *Winogradskyella* *tangerina* M1309^T^, *W. ursingii* HL4150 and MAG_Tara_SAT96 remained unaffiliated, as they formed independent single lineages. The phylogenetic groups of the “thalassocola” branch showed consistently larger genomic sizes in comparison with the atlantica lineage. PGII showed a genome size average of 4.57 ± 0.19 Mb, followed by PGI with 4.14 ± 0.28 Mb and PGIII with 3.69 ± 0.19 Mb. The genome size in the “atlantica” branch was smaller, with 3.38 ± 0.11 Mb on average for PGV and 2.16 ± 0.51 Mb for PGIV. All genomes with completeness higher than 95% were pairwise compared by reciprocal best matches and yielded a total of 13,154 orthology groups (OGs; Supplementary Table S3). All genomes shared 1,065 OGs (considered as the core-genome of the genus; Supplementary Table S4A) and a set of auxiliary genes of 12,089 OGs, of which 4,115 were genome-specific (Supplementary Table S4B). On the other hand, phylogenetic groups I–V shared 2051, 2433, 2075, 1719 and 1813 OGs, respectively.

### Putative polysaccharide and protein degradative capacity

**Fig. 2:**
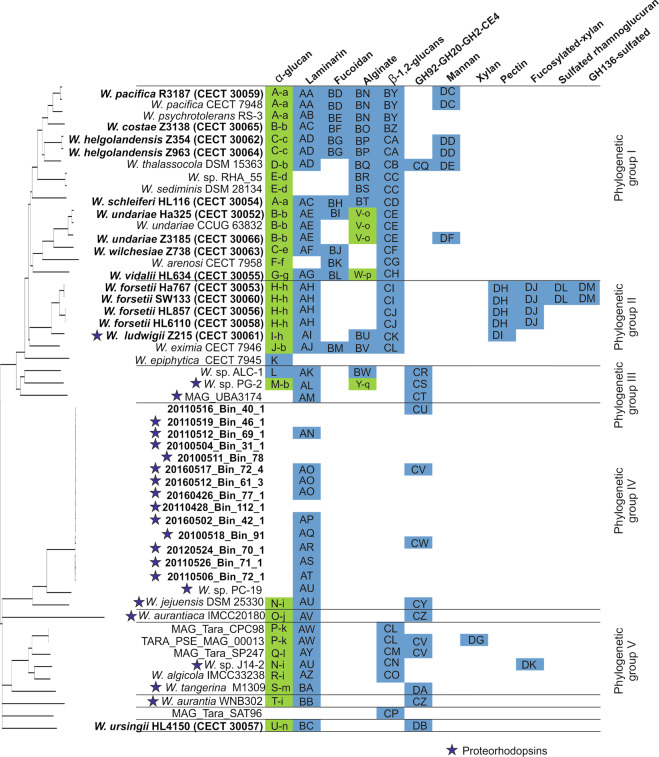
Consensus tree of all *Winogradskyella* genomes based on the Neighbor-Joining algorithm and predicted polysaccharide degradation capacities based on of PUL-associated CAZymes. Green means two PULs, blue means one PUL. Letters in each square mean exact gene arrangement of PULs. Strain deposit number is in brackets.

**Fig. 3:**
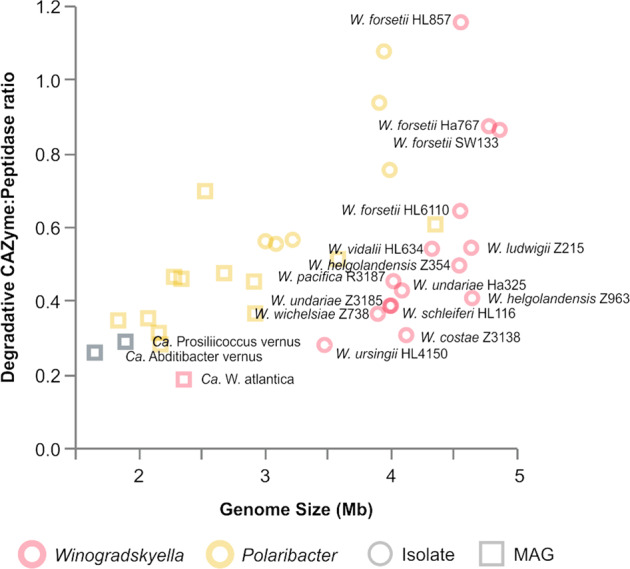
Plot comparing sizes (*x*-axis) and CAZyme/peptidase ratios (*y*-axis) of genomes and metagenome-assembled genomes of the genera *Winogradskyella* and *Polaribacter*. Circles indicate genomes, squares indicate MAGs. The two different colors distinguish the two genera.

The CAZyme occurrence showed an exponential increase in numbers with the increase of genome size (Supplementary Fig. S7A). On the other hand, the peptidase number of genes increased linearly with the genome size (Supplementary Fig. S7B). The degradative CAZymes (Supplementary Fig S7C) and predicted PULs (Fig. 2) in each genome suggested the potential capacity to degrade polysaccharides. The North Sea isolates belonging to the same species encoded for a similar number of CAZymes and peptidases independently of sampling time or the media of isolation (Supplementary Table S1). In general, the isolates encoded 44 to 90 CAZymes, with the exception of *W. forsetii* which encoded 113 to 202 CAZymes. In all cases, peptidases ranged from 129 to 175. In contrast, among *Ca*. W. atlantica North Sea MAGs more than 90% complete, the average numbers were 22 (±2) CAZymes and 127 (±3) peptidases. The ratio of CAZymes to peptidases across the *Winogradskyella* genomes as compared to the genus *Polaribacter* is shown in Fig. 3. Among species identifiable only from MAGs, it is clear that in general, and not restricted to the genus *Winogradskyella*, the trend is toward smaller genomes with fewer CAZymes per peptidase than among the larger isolate genomes.

The prediction of PULs (Fig. 2, Supplementary Fig. S8A–H) also allowed us to hypothesize on potential sugar target preferences of the phylogenetic groups. There was a larger and more diverse number of PULs in PGI and PGII, and PGIV encoded the lowest number of PULs. PULs for laminarin and alpha-glucans were present in almost all genomes, but conspicuously, all *Candidatus* W. atlantica MAGs, MAG_UBA3174 and MAG_Tara_SAT96 lacked the latter that were always present in all other members of *Winogradskyella* studied. The PUL “GH92-GH20-GH2-CE4” was unevenly found in all phylogenetic groups but in none of PGII. The alpha-mannosidase function typical of the GH92 family, and the hexosaminidase function of many GH20 family CAZymes would suggest some kind of algal cell wall polysaccharide may be the likeliest target of this gene cluster. Fucoidan and more obvious mannan PULs were only present in PGI; while alginate PULs were also present in PGII and PGIII. Isolates belonging to the same species encoded the same repertoire of PULs, and also with the same exact gene arrangement; (Supplementary Fig. S8A–H). The only exceptions were the two isolates *W. undariae* Ha325 and Z3185, which encoded a fucoidan and a mannan PUL, respectively, that were missing in the type strain CCUG 63832^T^. Both detected genotypes of *W. forsetii* shared all targets but genotype I (Ha767 and SW133) encoded for two extra PULs of sulfatedrhamnoglucan and an otherwise enigmatic PUL containing GH136 family CAZymes and sulfatases, for which an obvious polysaccharide substrate cannot be identified.

### Comparison of the gene repertoire of the isolates and MAGs

The most relevant genomic features to highlight were genes encoding proteorhodopsins and gliding motility. Proteorhodopsin was absent in all members of the PGI-III, excepting *Winogradskyella* *ludwigii* Z215^T^ (PGII), MAG_UBA3174 and *Winogradskyella* sp. PG-2 (PGIII). On the other hand, most members of the “atlantica” branch encoded for this feature, with the exception of Bin_40_1, the four MAGs from the Tara Oceans, *W. algicola* and *W. ursingii* HL4150 (Supplementary Annotation Data S1). The gliding motility observed for the North Sea isolates [14] was confirmed by the presence of all required genes for this feature (Supplementary Annotation Data S2) with the exception of *W. costae* Z3138^T^, which lacks the *sprE* gene. On the other hand, the *Ca*. W. atlantica MAGs Bin_69_1, Bin_42_1 and Bin_70_1 encoded the full essential set of genes required for gliding, and one or more of these genes were missing in the remaining MAGs.

One additional outstanding observation was that all genomes encoded the aerobic enzyme cytochrome c oxidase, but the genes for the microaerophilic enzymes cytochrome bd ubiquinol oxidase were missing in PGIII and IV in the former and the cytochrome ccb3-type in PGIV for the latter (Supplementary Annotation Data S3). The *bapA*, a biofilm related protein, was encoded in only one copy in PGIV, while several copies (ranging from 2 to 8) were present in the rest of phylogenetic groups. All PG encoded a catalase-peroxidase (*katG*), but PGIV lacked the catalase (*katE*) which was present in all other PG. Many genes involved in amino-acid synthesis (arginine, methionine, leucine and valine) were found missing for PGIV, while a greater number of protein and amino-acid degradation genes were found in PGIV than the rest of the groups. In addition, the genes involved in nitrogen assimilation such as nitrilases were only present in PGIV. All PG had one copy of each the glutamate synthase chain (*gltB, gltD*), which was missing in all the “atlantica” branch (PGIV-V) with the exception of *W. aurantiaca*, *W. tangerina* and MAG_Tara_SP247. The glutamine synthetase (*glnA*) was present in two copies in all genomes, but only in one copy in PGIV. The glutaminase (*glsA*) was present only in PGI and PGII, while these two groups both had two copies of a glutamate dehydrogenase (GDH), as did the TARA_PSE_MAG_00013 and *W. ursingii*. Interestingly, variable genes involved in sulfur assimilation were found across the genomes, but only *Candidatus* W. atlantica Bin_112, Bin_69 and Bin_72_1 contained sulfur-related genes such as the *cysC, cysN, cysD* and a sulfate permease.

Finally, we checked genetic domains related to anaplerotic carbon fixation. Proteorhodopsin containing *Winogradskyella* had a higher number of these genes per Mb (Supplementary Annotation Data S4) which has been related to a free-living lifestyle [27].

### Genomic comparison within PGIV

The closest relatives of *Ca*. W. atlantica were *W. jejuensis* and *Winogradskyella* sp. PC-19. The two strains exhibit the smallest genomes among the currently available isolates of *Winogradskyella*, with 3.03 and 2.98 Mb, respectively. *W. jejuensis* was isolated from seaweed tissues while *Winogradskyella* sp. PC-19 has a seawater origin.

All PGIV genomes (>90% completeness) shared 2,045 OGs. Some 700 OGs and 733 OGs were present in *W*. sp. PC-19 and *W. jejuensis*, respectively, encoding additional functions absent in the *Ca*. W. atlantica genome (Supplementary Annotation Data S5). Some traits present in both isolates were the redox involved protein thioredoxin 1 (*trxA*), the starvation-inducible DNA-binding protein (*dps*), several amino-acid synthesis related genes such as *metF* and *metH*, the porin OOP family, the lactoylglutathione lyase (GLO1) which has been described as an enzyme contributing in the survival of *Salmonella* in nutrient rich environments [69], and the aforementioned cytochrome c oxidase *cbb3*-type microaerophilic enzyme. Furthermore, *Ca*. W. atlantica also showed fewer duplicates of genes present in the other genomes such as the TonB-related periplasmic proteins, two-component system transcriptional regulators, ABC-transport systems, *lacI* family transcriptional regulators or iron complex outer membrane receptor proteins among others.

### *Winogradskyella* genomes and MAGs in the metagenomic data

**Fig. 4:**
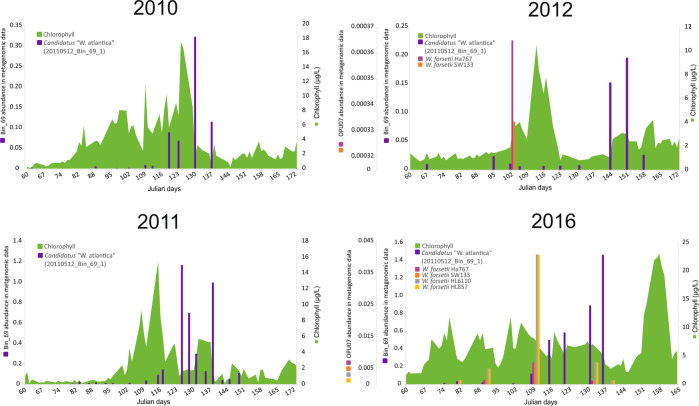
Estimation of reference *Candidatus* “W. atlantica” Bin_69_1^T^ and *W. forsetii* (OPU07) abundances during spring phytoplankton bloom of Helgoland in years 2010–2012 and 2016 (Supplementary Table 5) based on reads proportion of the 47 metagenomic datasets recruited to the different *Winogradskyella* genomes. Years 2012 and 2016 have two different scales for genomic abundance.

The metagenomic time-series allowed us to evaluate the presence and abundance of the genomes along 2010–2012 and 2016 (Supplementary Table S5). North Sea MAGs were recurrently detected during the spring bloom in all four studied years. The *Ca*. W. atlantica Bin_69_1^T^ was chosen to represent the genomospecies (Fig. 4), but all MAGs followed the same trends in terms of abundances (Supplementary Table S5). Bin_69_1^T^ was most abundant from late April to mid-May (Julian days ~ 115 to 140 during years 2010, 2011 and 2016; Fig. 4), and showed the highest abundances in 2016 with 1.46% of the total read abundances. On the other hand, the overall lowest abundances were found in 2012 with a maximum 0.19%, and also appeared later in the season (~ Julian days 144–158).

From the cultured species, we could only detect the presence of *W. forsetii* (Fig. 4) in low numbers ranging up to a maximum of 0.04% of the total reads (Supplementary Table S5). From it, the genotype I (Ha767 and SW133) was detected only once in 2012, and in three samples of the 2016 time series. The abundances found in 2016 were between 2 and 18 times higher than in 2012. Genotype II (HL6110 and HL857^T^) was found in the same three samples as genotype I, as well as in two additional samples in 2016. No other isolate genomes were confirmed to be present in the dataset, although some reads could be recruited but abundance not quantified due to low coverage of the genome (<0.001%; Supplementary Fig. S9), and although this is most likely non-specific 16S rRNA gene mapping of a small number of reads (Supplementary Table S5).

### 16S rRNA gene MED nodes

**Fig. 5:**
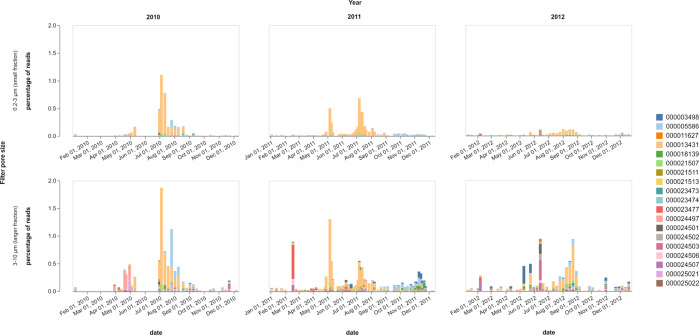
Relative 16S rRNA gene read abundances of *Winogradskyella* nodes in surface seawater fractions 0.2–3 μm and 3-10 μm during years 2010 to 2012. Node 13431 is expected to represent the same species as *Candidatus* “W. atlantica” Bin_69_1^T^.

16S rRNA gene amplicon sequencing was performed for the same environmental DNA samples obtained in the years 2010–2012 [65]. Amplicons were clustered using MED to better identify stable patterns of diversity. The two distinct filtered fractions 0.2–3 μm and 3–10 μm yielded 19 different *Winogradskyella* MED nodes [65]. *Winogradskyella* was detected in recurrent blooms during the spring and summer seasons and remained at low abundance during the winter, with a seemingly distinct set of populations present in the period October - December 2011 (Fig. 5). The predominant node during all years sampled in both fractions was the same (ID: 13431) and reached its maximum of 1.03% in the 0.2–3 µm fraction and 1.25% in the 3–10 µm fraction in July of 2010 (Supplementary Table 6). Node 13431 and Bin_69_1^T^ are expected to represent the same *Ca*. *W. atlantica* population, given the near identical patterns of read abundance between MED nodes and MAGs. However, the absence of 16 S rRNA gene sequences in the MAGs prevented a correlation with the MED node sequences. A second more abundant node, node 5586, was also found in the smaller size fraction. Overall diversity was lower in the smaller fraction, which was dominated by node 13431, followed by node 5586 (Fig. 5). In contrast, the larger fraction showed a higher diversity of sequences, although the relative abundance of these was low in comparison to that of the putative *Ca*. W. atlantica.

## Discussion

The isolation of members of *Winogradskyella* in a high-throughput survey from one well-studied ecological situation, the spring bloom in the North Sea [15], provided the opportunity to explore the ecological relevance and genomic traits of species in this genus. Phylogenetic analyses revealed two major lines of descent. A larger clade comprised the type species of the genus (*W. thalassocola*), most of the 42 species of the genus, and most of the novel species from the North Sea. The second, smaller, clade included most of the MAGs detected in environmental surveys, including *Ca*. W. atlantica, the abundant species from the North Sea spring bloom [14]. The sequenced genomes of the *Winogradskyella* clade *sensu stricto* (the “thalassocola” branch) had larger genomes, with a more complex genetic repertoire including a diverse range of PULs and proteases. This feature has been identified in algae-associated (particle-attached) *Bacteroidetes* [28, 70]. In addition, the biofilm-associated protein gene (*bapA*) and the presence of the microaerophilic enzymes genes (*cydA-cydB* and *ccoNO-ccoP*) indicate the potential to live in microaerobic biofilms on particles. A complete set of defense enzymes against reactive oxygen species provides the capacity to live in the light on algae and outside of the aqueous phase on plates, where the oxygen flux to cells is greater than in water. The genomes of the “thalassocola” branch clearly provide the means to live as a generalist, adapting to a variety of challenging environmental conditions.

In contrast, smaller genomes characterize the sequenced members of the “atlantica” branch, with that of *Ca*. W. atlantica the smallest. Environmental nutrient resources during the decay of the spring diatom bloom are the cytosolic content of the algae, with laminarin as the major soluble carbon storage molecule [71–73]. *Ca*. W. atlantica has only one PUL in its streamlined genome, predicted to target laminarin. This species lacks the capacity to use many different polysaccharides, and with its smaller genome it has a lower biosynthetic constraint on replication and proliferation. The bloom of the species during spring algal decay demonstrates that this presumed speciation resulted in a bacterium capable of competing with other laminarin specialists such as *Polaribacter* species. As laminarin does not provide reduced nitrogen and sulfur compounds for biosynthesis and proliferation, the genome contains a range of proteases that would supply the source of nitrogen and sulfur. Living freely in the water column reduces selective pressure to maintain enzymes for attachment, microaerobic respiration, and a high defense capacity against reactive oxygen species. In line with this ecological niche is the lack of an identifiable catalase in all the metagenome-derived genomes of the “atlantica” branch. Furthermore, algal biomass provides access to many metabolites (building blocks for polymers and vitamins) so that the genome of *Ca*. W. at**l**antica might have been further streamlined in this respect than even those of the SAR11 clade and other oligotrophic free-living bacteria. The lack of an identified pathway for proline synthesis is a first piece of corroborating evidence, as essential amino acid biosynthetic pathways have been annotated in other streamlined genomes [34, 74, 75]. Thus, genomic streamlining of free-living bacteria in the planktonic zone of the ocean may lead not only to a specificity toward the preferred anabolic substrate, but also to specific auxotrophies for available nutrients such as nitrogen and sulfur compounds, as well as cofactors (vitamins) [76]. This specialization can explain the ephemeral recurrence of *Ca*. W. atlantica with seasonal phytoplankton blooms, while demanding survival during the other times of the year. Proteorhodopsin and the high affinity of the *TonB*-dependent outer membrane selfish-uptake system for laminarin [25] may be essential for this survival. Indeed, it has been proposed that *Bacteroidetes* have adapted to life in oligotrophic surface waters by small genomes containing proteorhodopsins in combination with a high potential for anaplerotic carbon fixation [27].

A large cultivation effort with the analyses of over 5000 strains yielded 40 *Winogradskyella* strains of the “thalassocola” branch. The failure to obtain strains of *Ca*. W. atlantica or more generally of many of the abundant free-living planktonic bacteria as colonies on plates, the so-called great plate anomaly [77] is, in light of our analyses, more understandable. Studies on the methylotrophic clade OM43 [75] and the heterotrophic *Pelagibacter* clade [33] show unusual nutritional necessities resulting from genome reduction. A credible explanatory factor is the absence of biosynthetic pathways for essential nutrients. Colonies may form only when these metabolites are present in the growth media and are chemically stable during the growth period. Substrate-induced cell death may also contribute to the lack of cultivability in the case of *Ca*. W. atlantica, which lacks the PII system, a metabolic regulatory system that prevents the uncontrolled influx of ammonium beyond cellular requirements. Many free-living planktonic bacteria, including SAR11 clades [78], lack this system and are apparently not cultivable on plates. In liquid culture, only limited amounts of ammonium are tolerated, as demonstrated for example in *Formosa*-affiliated strains [79]. Colony formation requires millimolar concentrations of nutrients, however only micromolar concentrations are present in the sea, allowing the removal of detoxification systems in the process of genomic streamlining. The lack of an identifiable catalase in the *Ca*. W. atlantica genome is another example of the absence of well-known bacterial protective systems in free-living planktonic bacteria with streamlined genomes. These bacteria seem to be cultivable only in liquid media at environmental or slightly elevated nutrient concentrations [79, 80]. The resulting cell density may reach ten million cells per ml, insufficient to form turbid cultures, but sufficient to explore the metabolic capabilities of the cells by genome, transcriptome, proteome and metabolome analyses. For improving cultivation success of species with reduced genomes, such as the *Ca*. W atlantica, culture media mimicking environmental conditions with respect to the parameters mentioned above or containing additional defense systems could have the potential to succeed in isolating the candidate taxa. In the future, strains with defined gene deletions may contribute to a better understanding of the physiological importance of genes that are present in the isolates and absent in the MAGs.

While we cannot unequivocally claim to have identified any one key reason for the evident discrepancy in both environmental abundance and cultivation success for the North Sea *Winogradskyella* species studied, the analysis we present here nevertheless provides options for consideration. Deficits in our understanding of microorganisms’ metabolic needs will need to be overcome if we wish to supplement insights gained from cultivation-independent surveys with culture-based investigations that would allow us to more rigorously test our hypotheses.

## Supplementary information

Supplementary Figures

Supplementary Tables
