# Supplementary material for: Cultivable Winogradskyella species are genomically distinct from the sympatric abundant candidate species
Source: ISME Commun. 2021 Sep 29;1:51. doi: 10.1038/s43705-021-00052-w (PMC9723794; doi:10.1038/s43705-021-00052-w)
Supplement: Supplementary file 1 — Supplementary Figures [file 43705_2021_52_MOESM1_ESM.docx]

**Supplementary Figure S1:** RAPD fingerprint patterns example of an agarose gel electrophoresis of random amplification of polymorphic DNA (RAPD) PCR amplicons from selected strains. The selection of strains to be genome sequenced was carried out considering different patterns to avoid clonality.


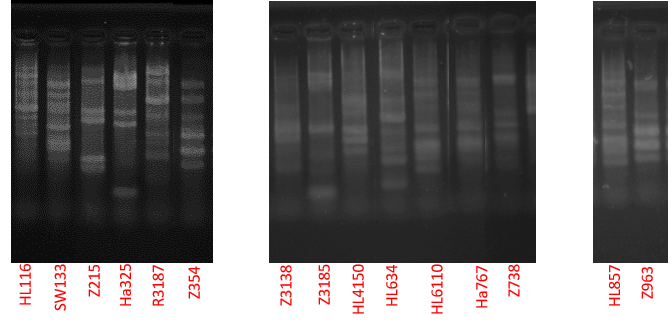


**Supplementary Figure S2:** A) Genomic clustering based on Average Nuclotide Identity (ANI) dendrogram of *Winogradskyella* genomes included in the study. Bin_69 as representative of all fifteen North Sea Bins B) Genomic clustering based on ANI of North Sea Bins.


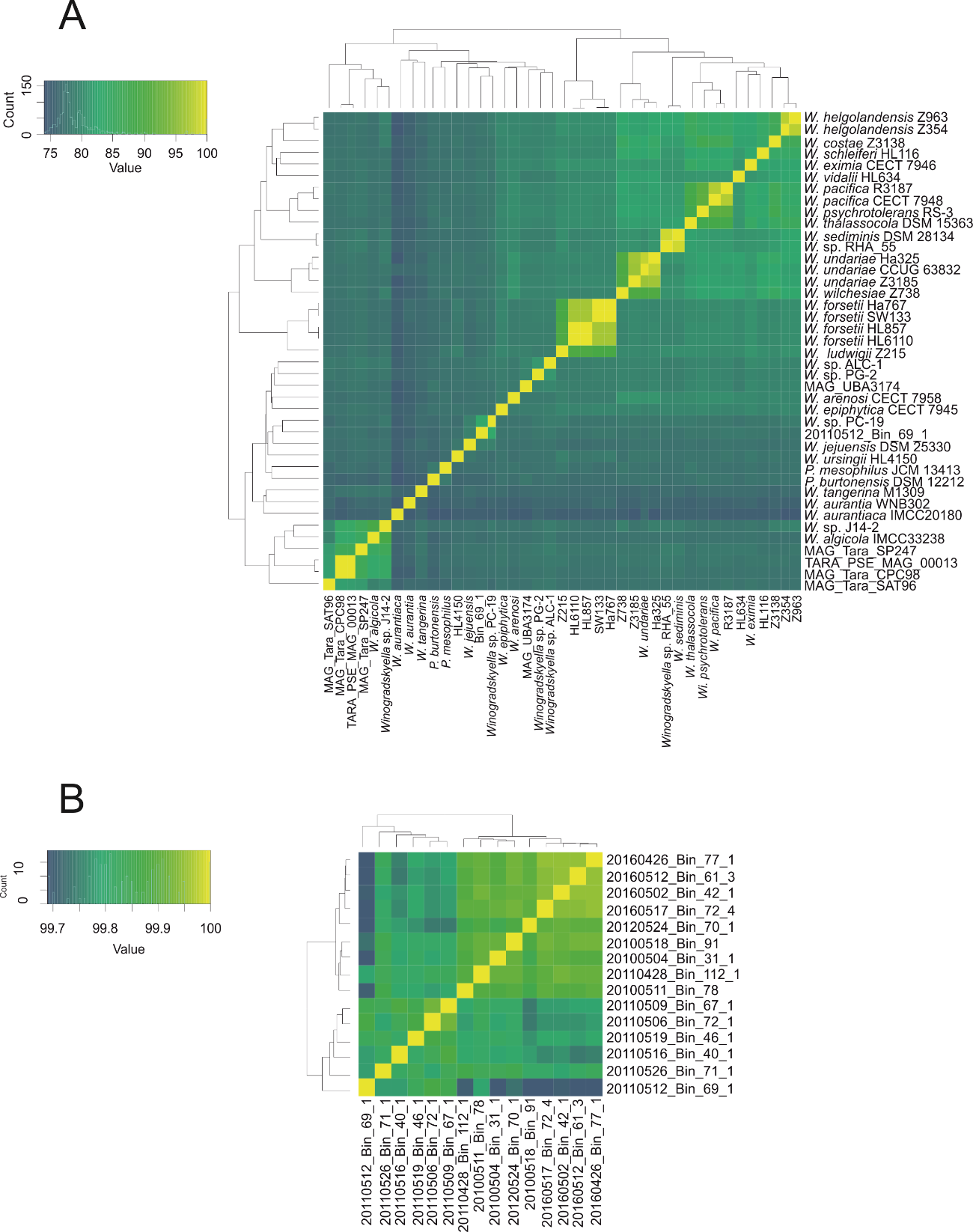


**Supplementary Figure S3:** A) Genomic clustering based on Average Amino-acid Identity (AAI) values (Sup. Table 2). Bin_69 is representative for all fourteen North Sea MAGs. B) ANI of North Sea Bins show they belong to same species, but three *Candidatus* “W. atlantica” MAGs recovered year 2011 (Bin 40_1, Bin_71_1 and Bin_46_1 may represent a different ecotype.


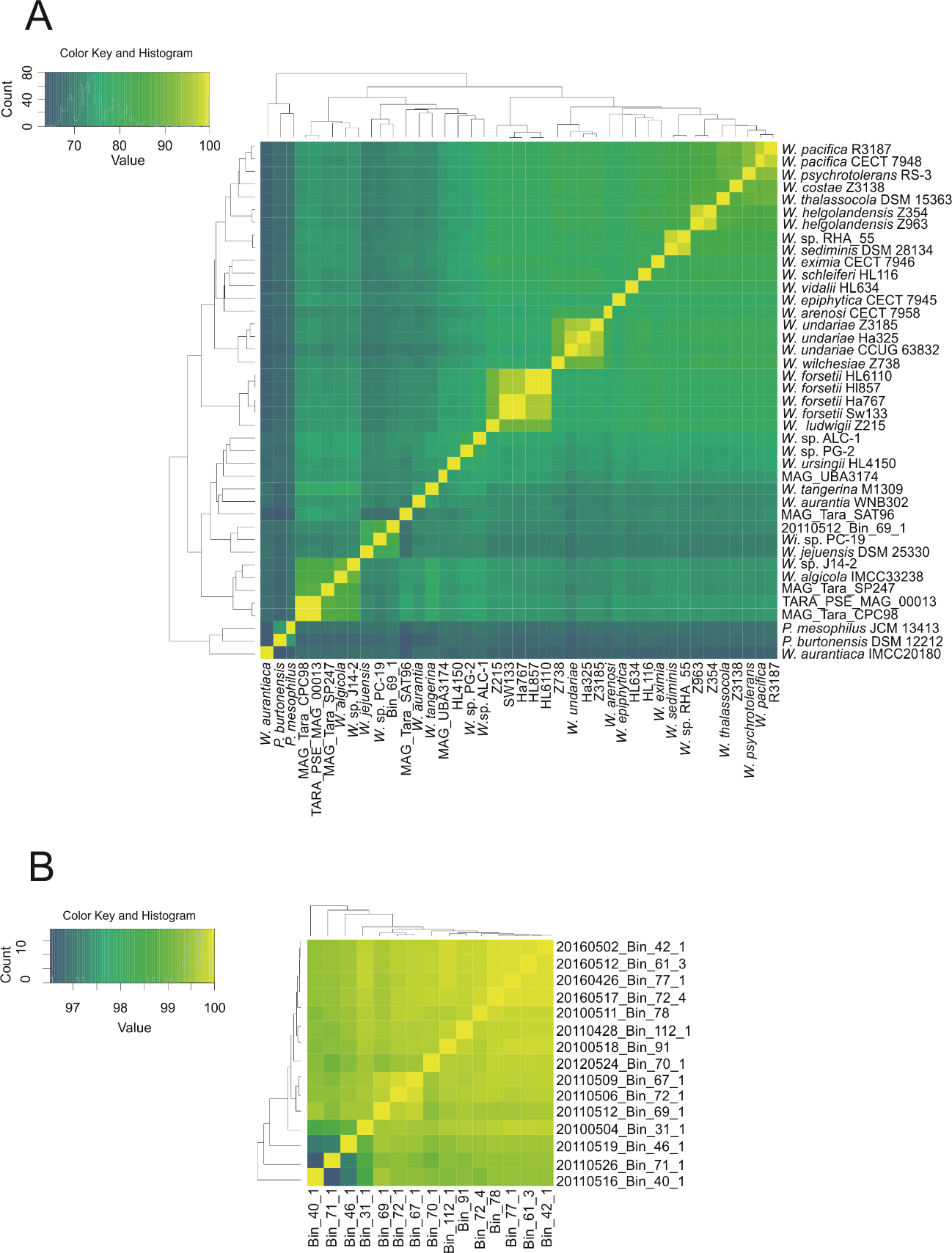


**Supplementary Figure S4:** Phylogenetic reconstruction of 16S rRNA sequences based on Neighbour-Joining (A no bootstrap, B bootstrap) and RAxML (C no bootstrap, D bootstrap); and consensus phylogenetic reconstruction (E) where branches not supported by all tree reconstruction algorithms are shown as multiforcations.


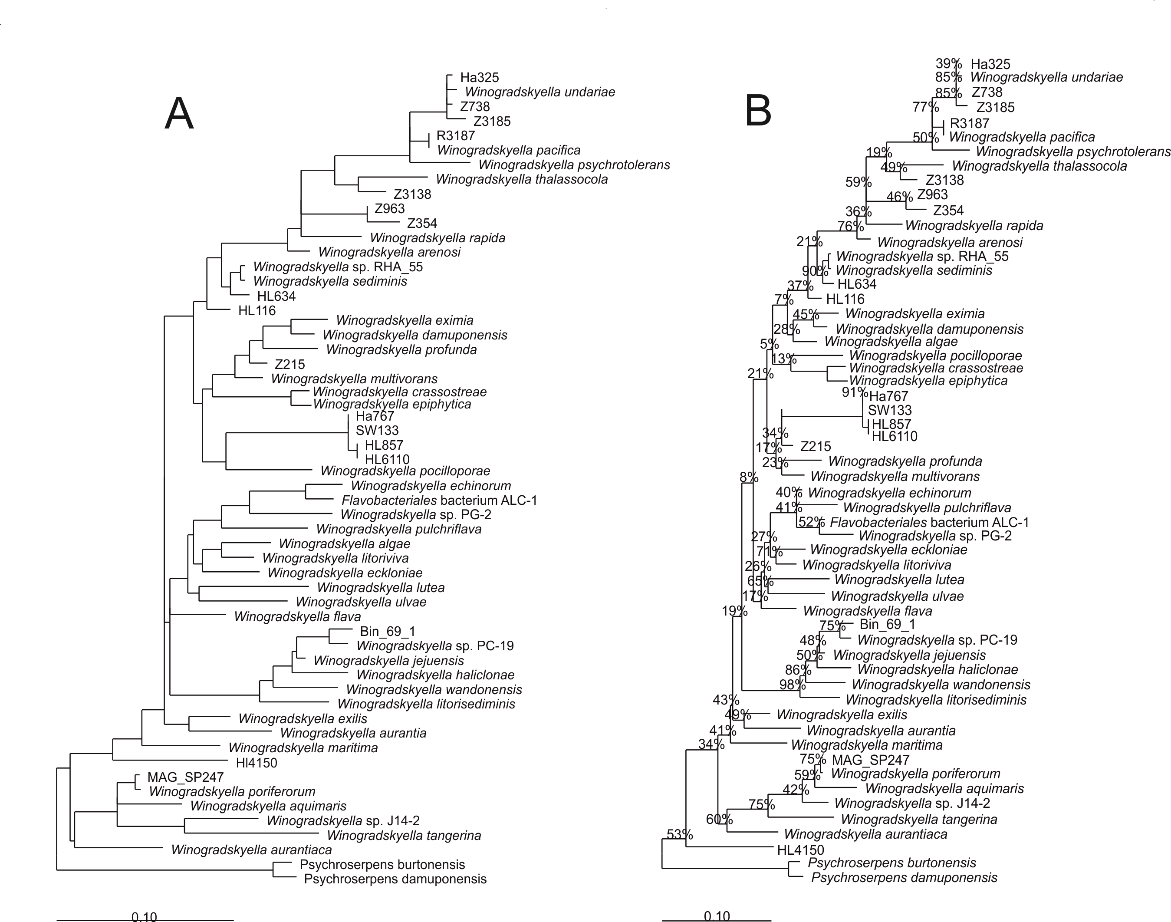


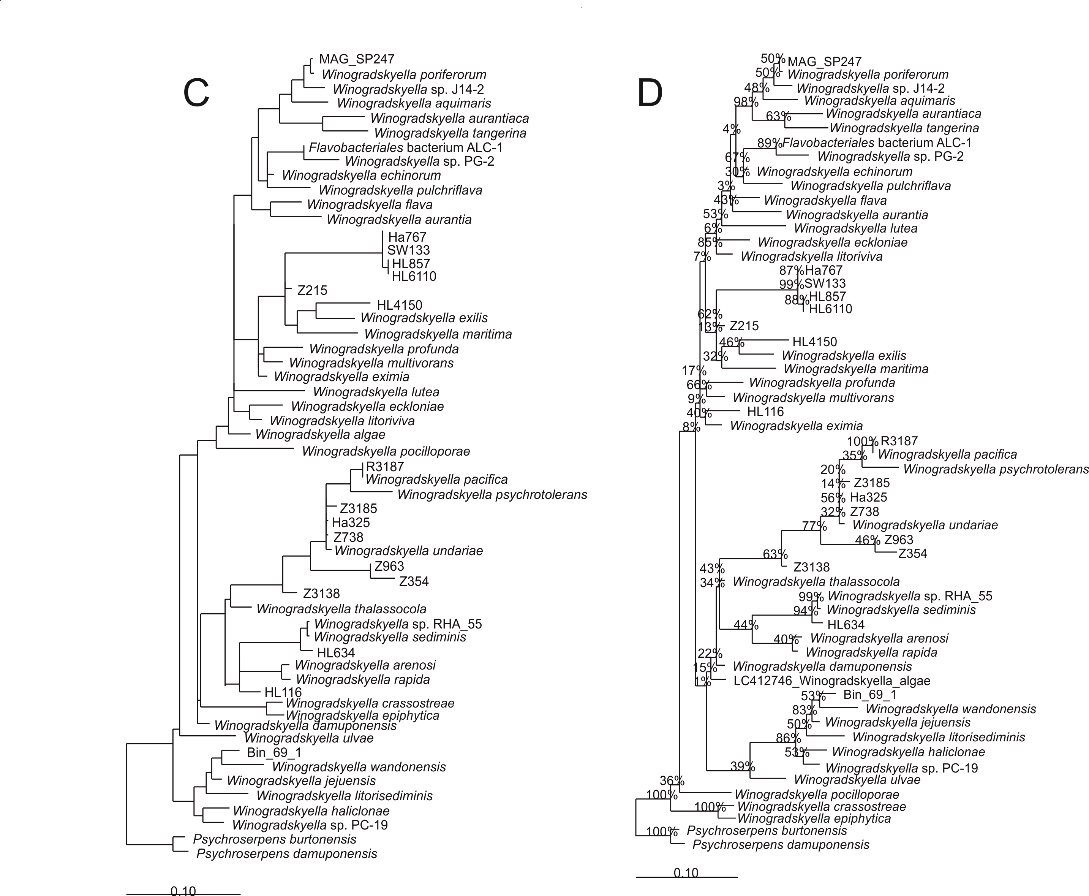


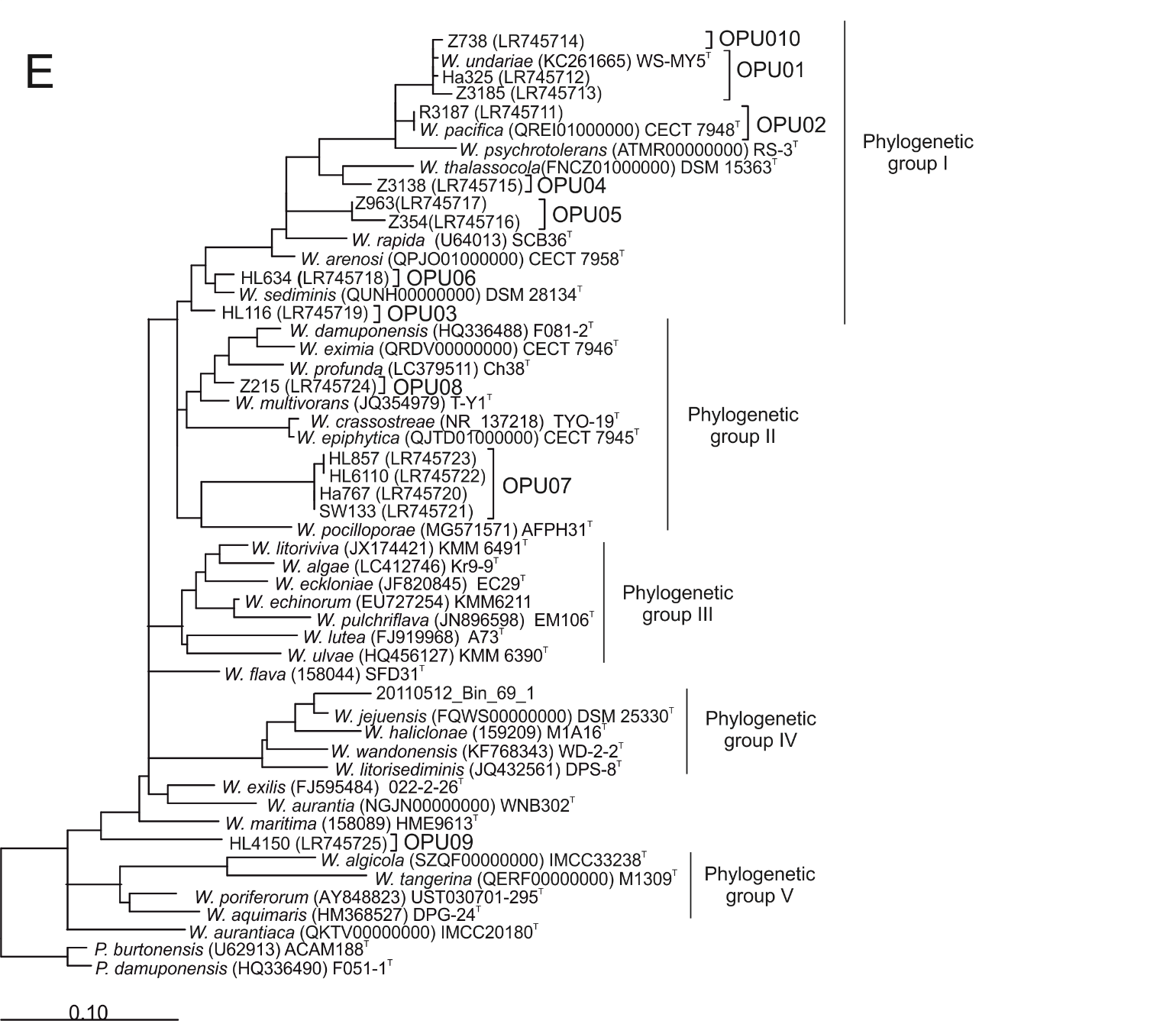


**Supplementary Figure S5:** A) Original phylogenetic reconstruction of twenty-one concatenated shared essential genes (core-essential genes) based on Neighbour-Joining algorithm B) Consensus phylogenetic reconstruction of core-essential genes where branches not supported by all tree reconstruction algorithms are shown as multiforcations.


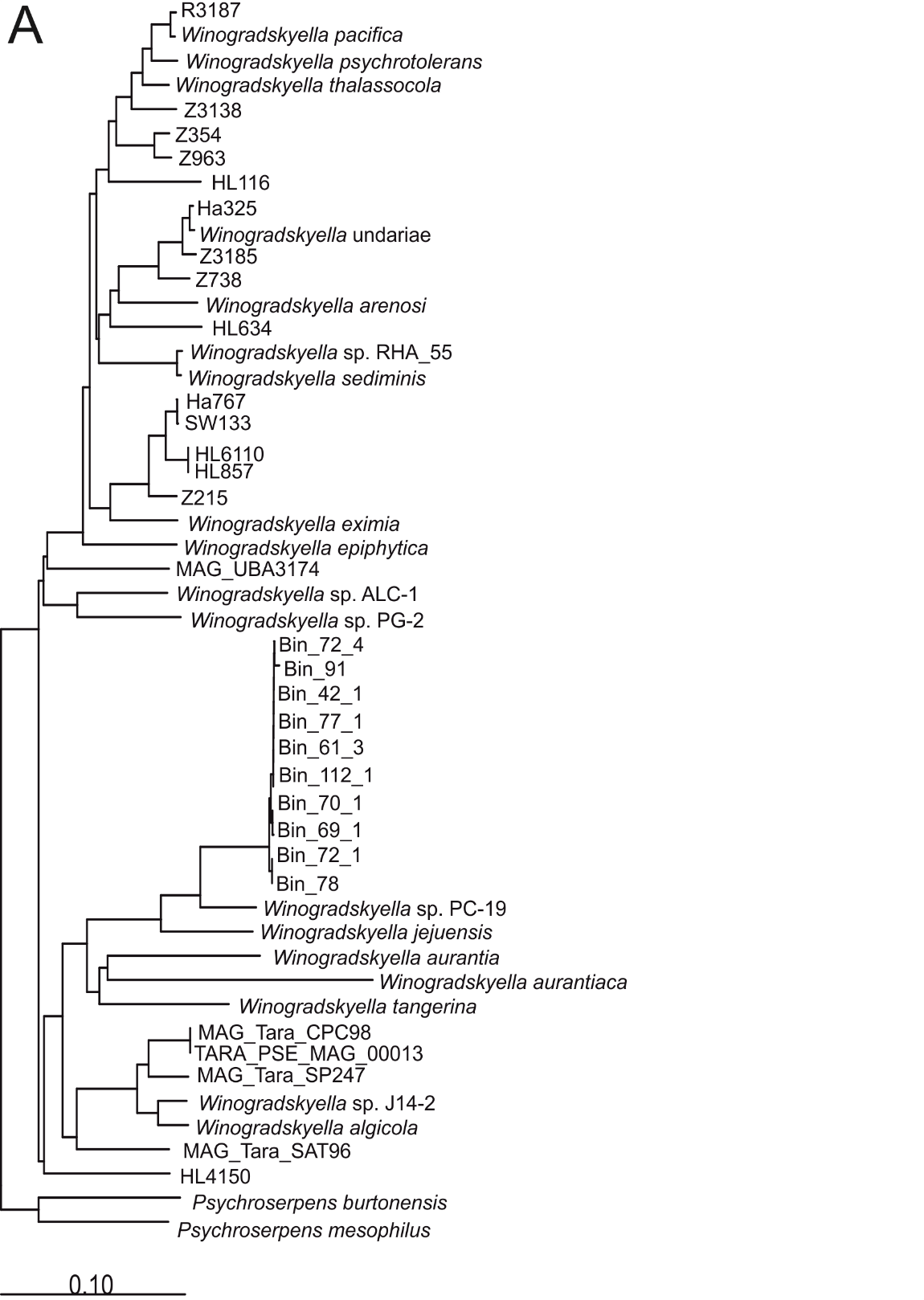


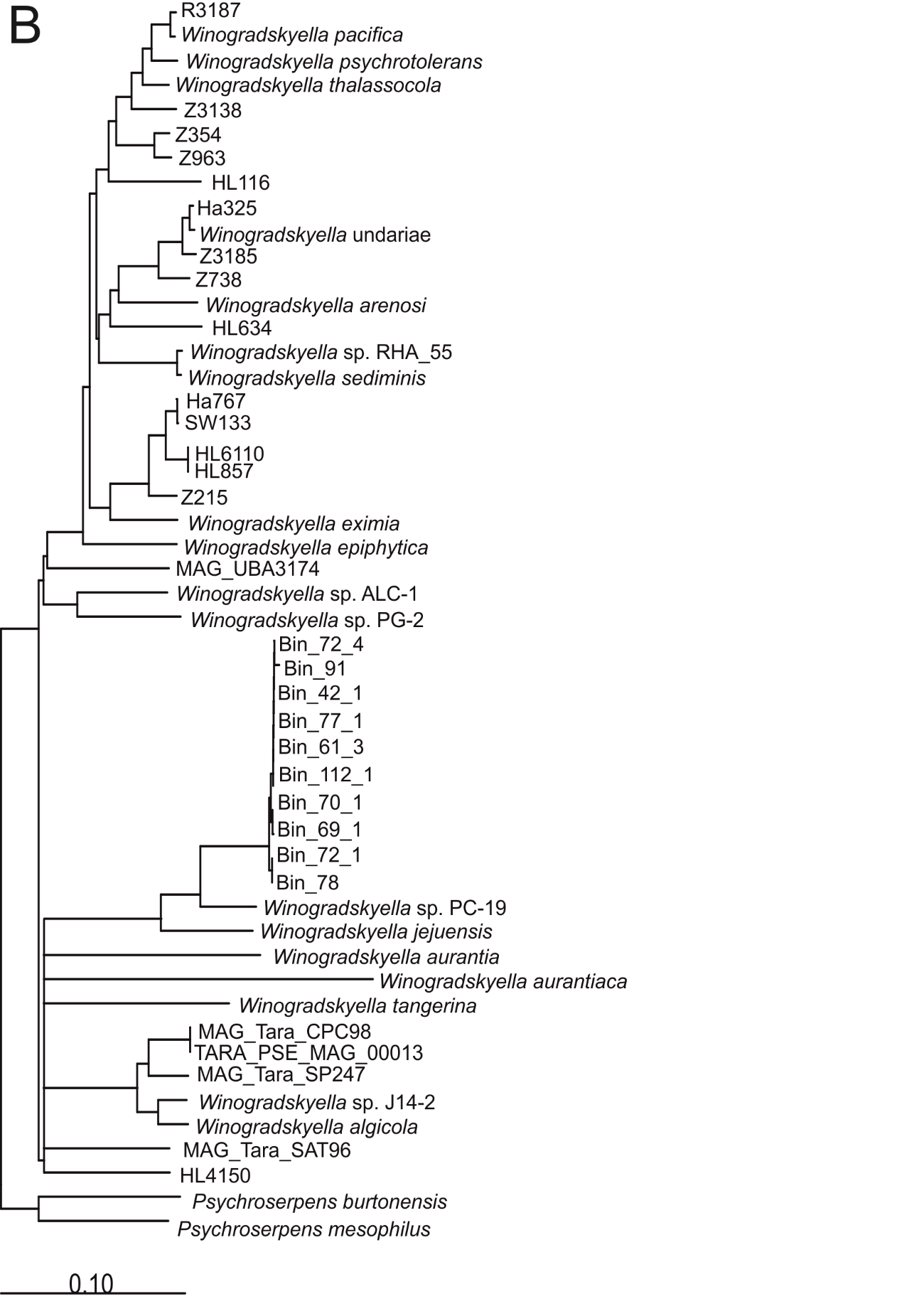


**Supplementary Figure S6:** A) Original phylogenetic reconstruction of the core genome based on Neighbour-Joining algorithm B) Consensus phylogenetic reconstruction of the core genome where branches not supported by all tree reconstruction algorithms are shown as multiforcations.


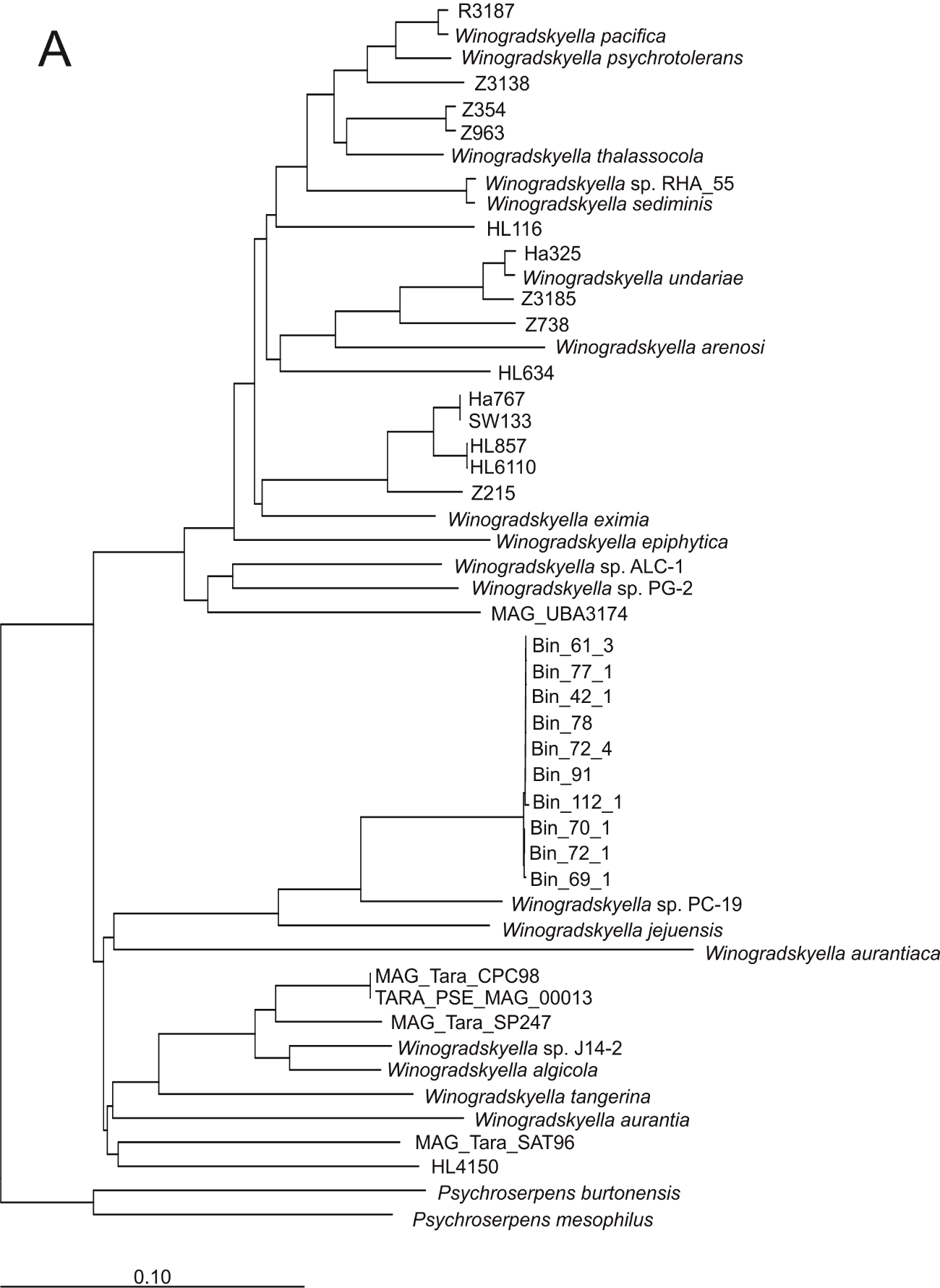


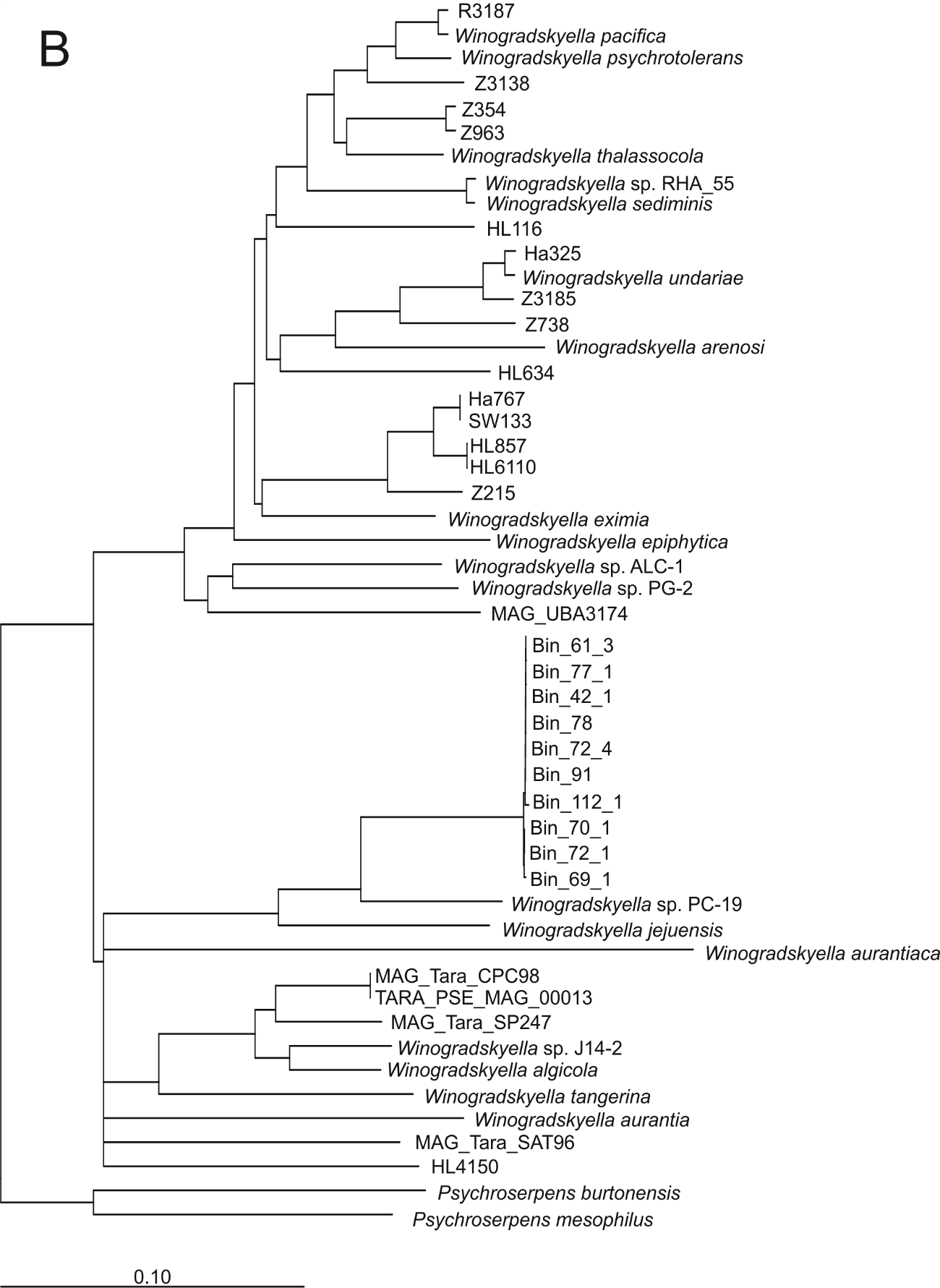


**Supplementary Figure S7:** Densities of (A) CAZymes with degradation functions vs genome size and (B) peptidases vs genome size, and (C) CAZymes:peptidase ratio.


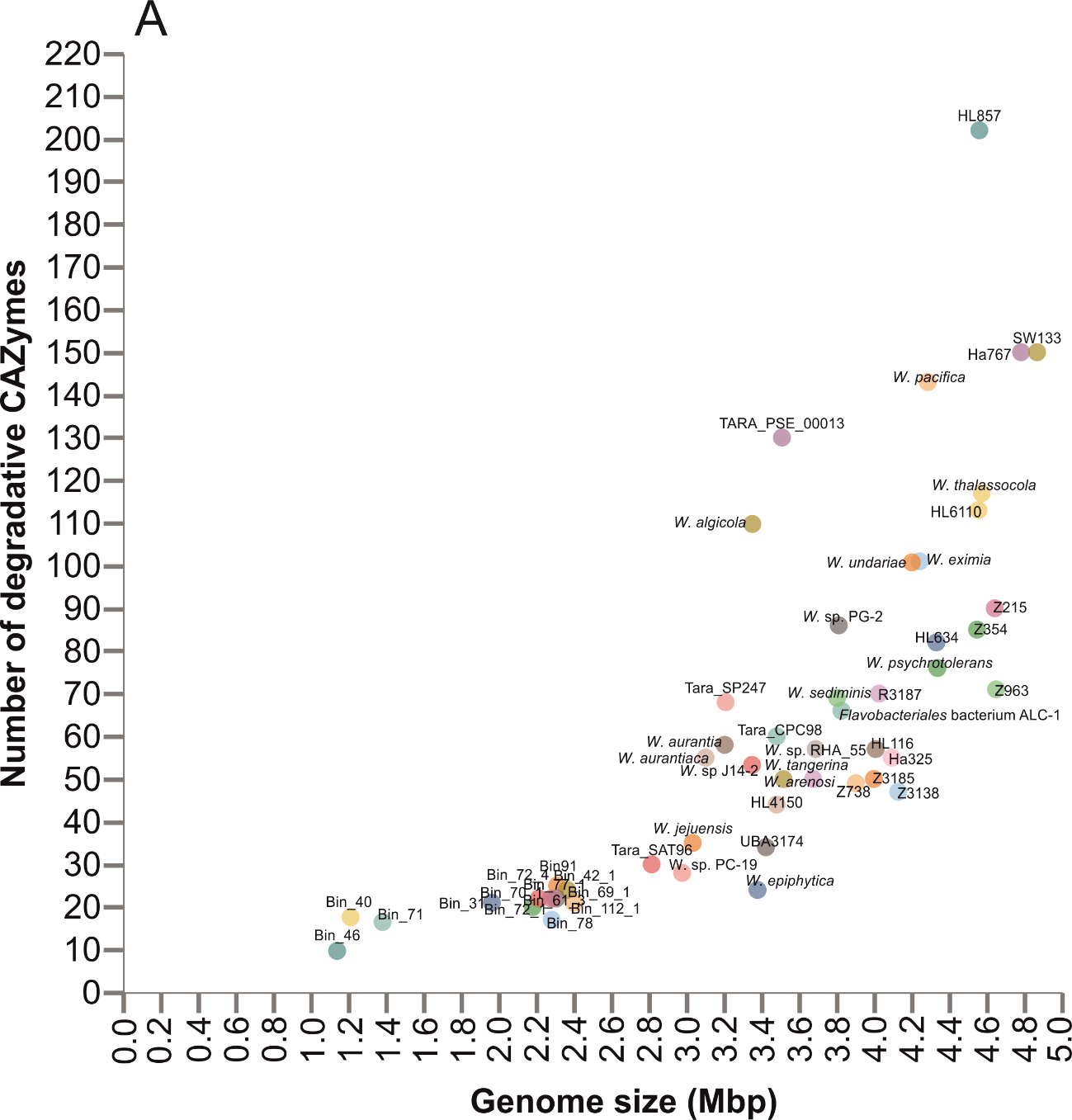


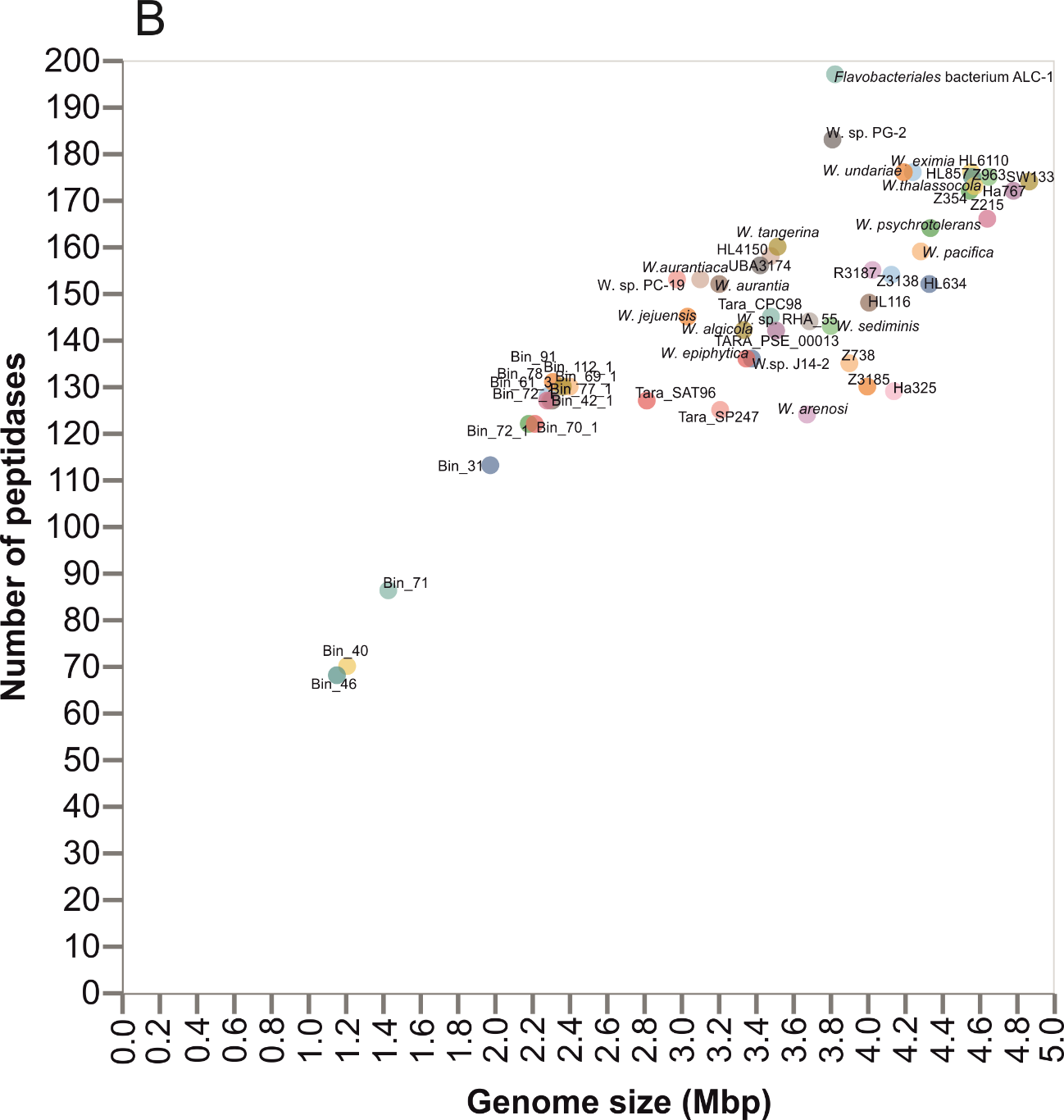


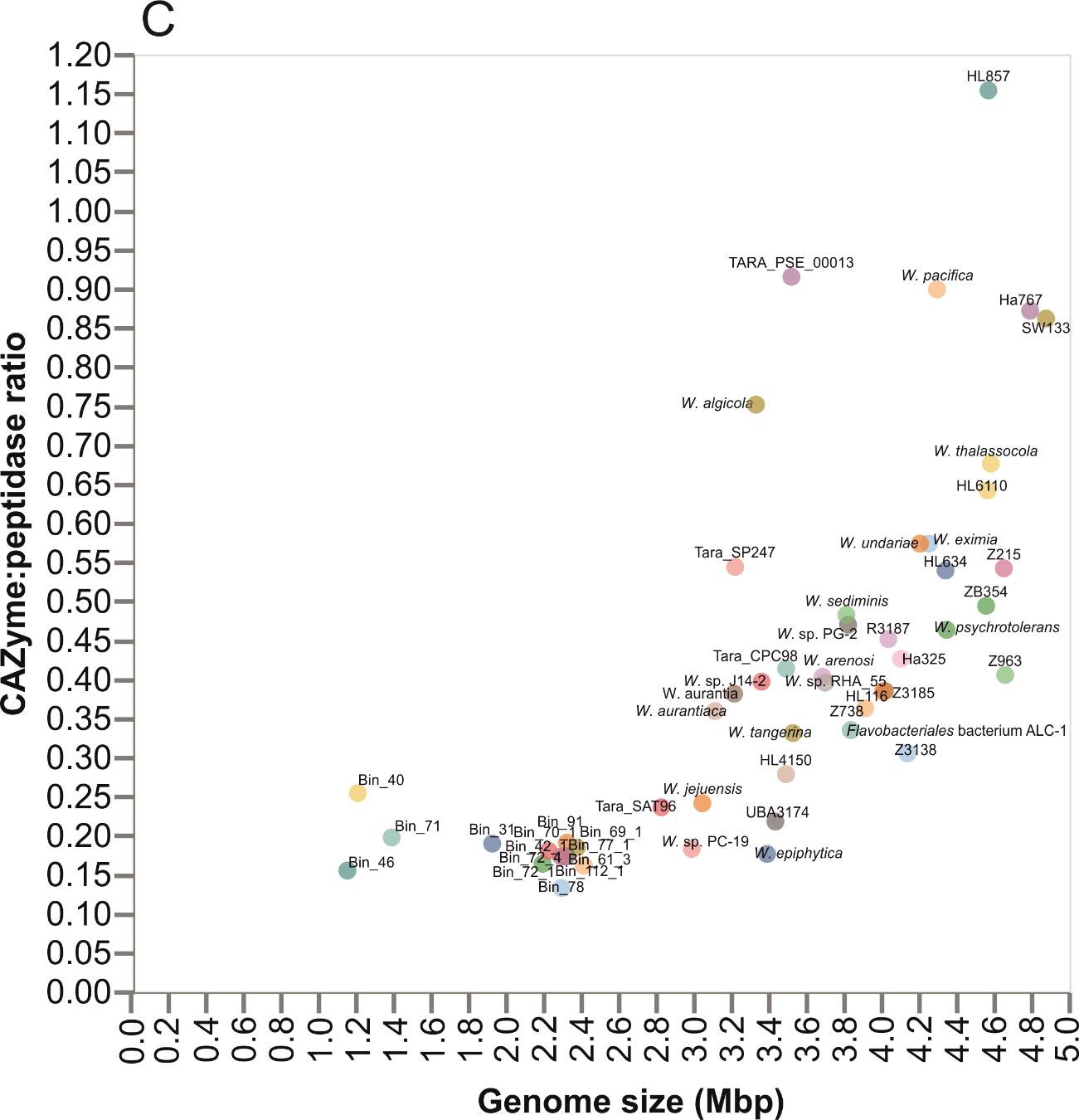


**Supplementary
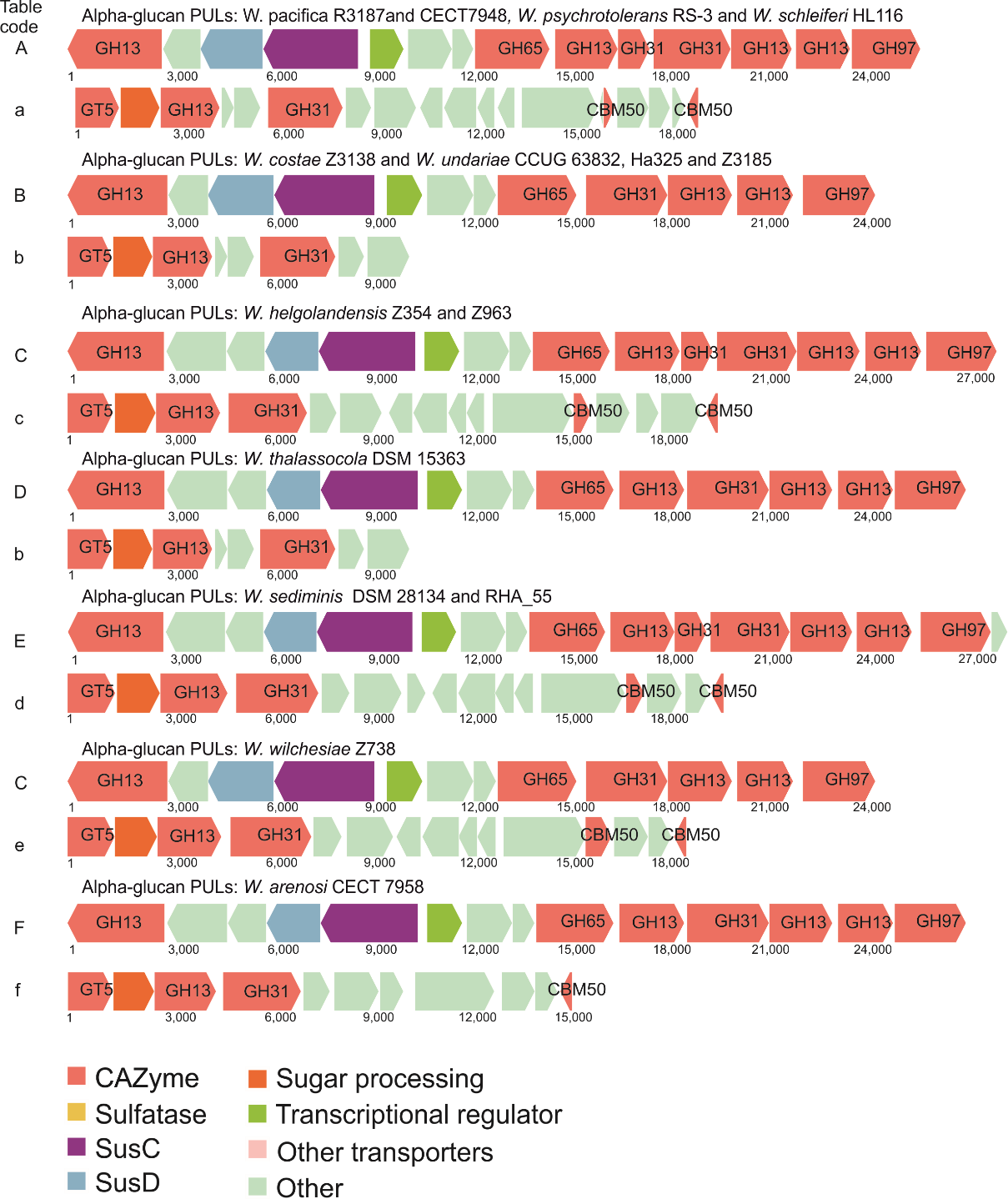
Figure S8A:** Alpha-glucans PULs of the *Winogradskyella* genomes in the study (all located on the larger of the two contigs). umbers in genes indicate family affiliations of glycoside hydrolases (GH), glycosyltransferases (GT) and carbohydrate-binding modules (CMB).


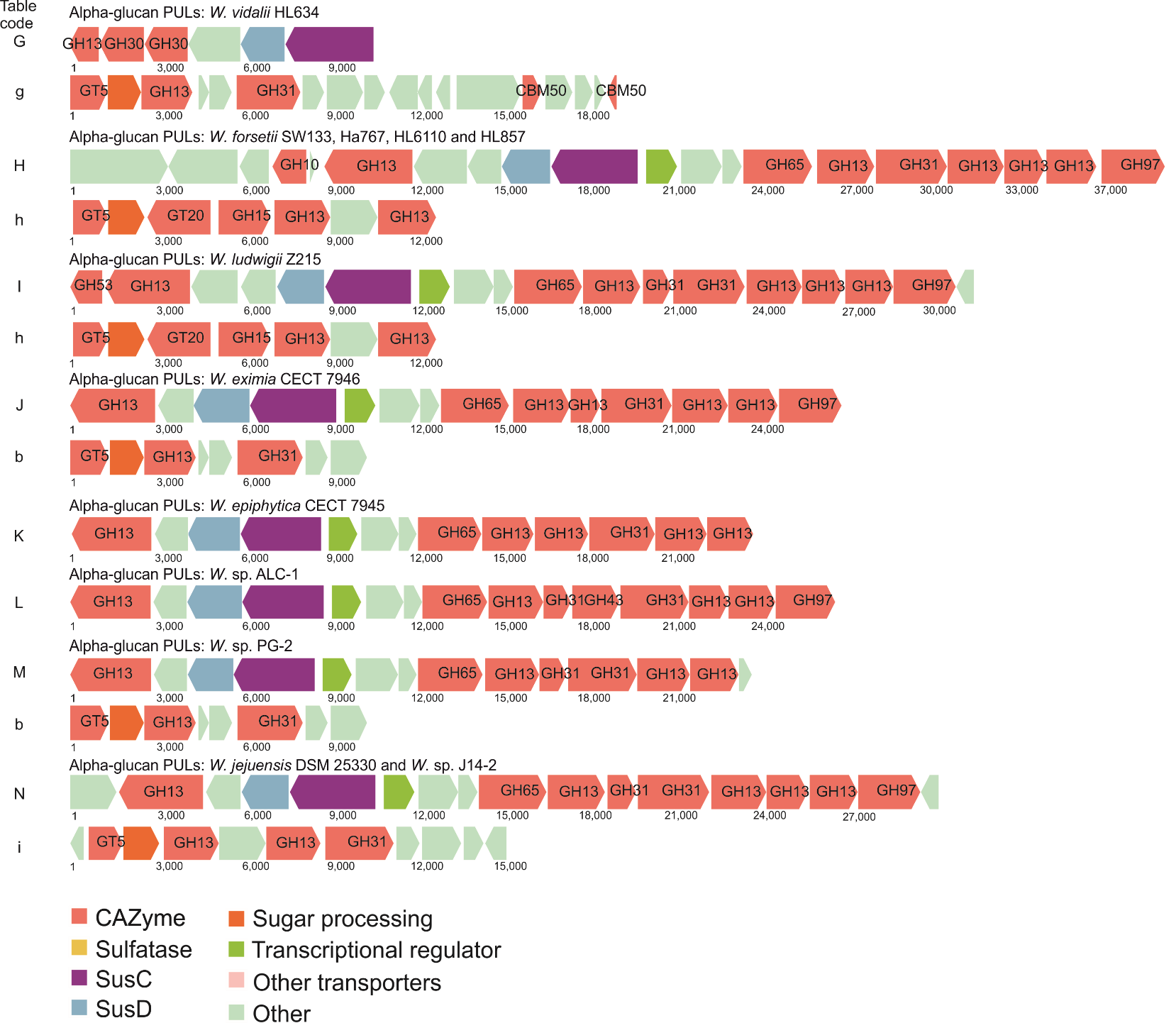


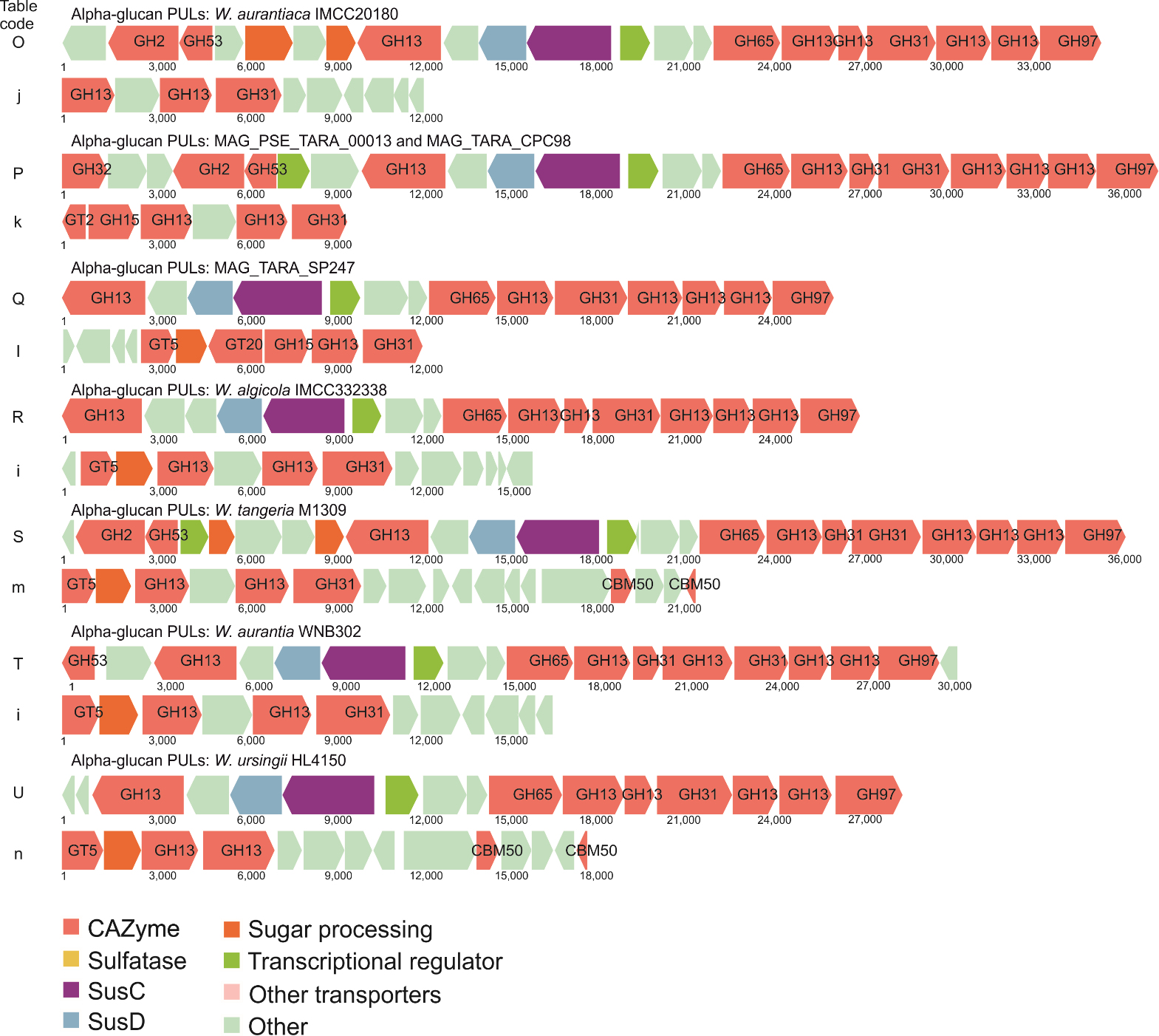


**Supplementary
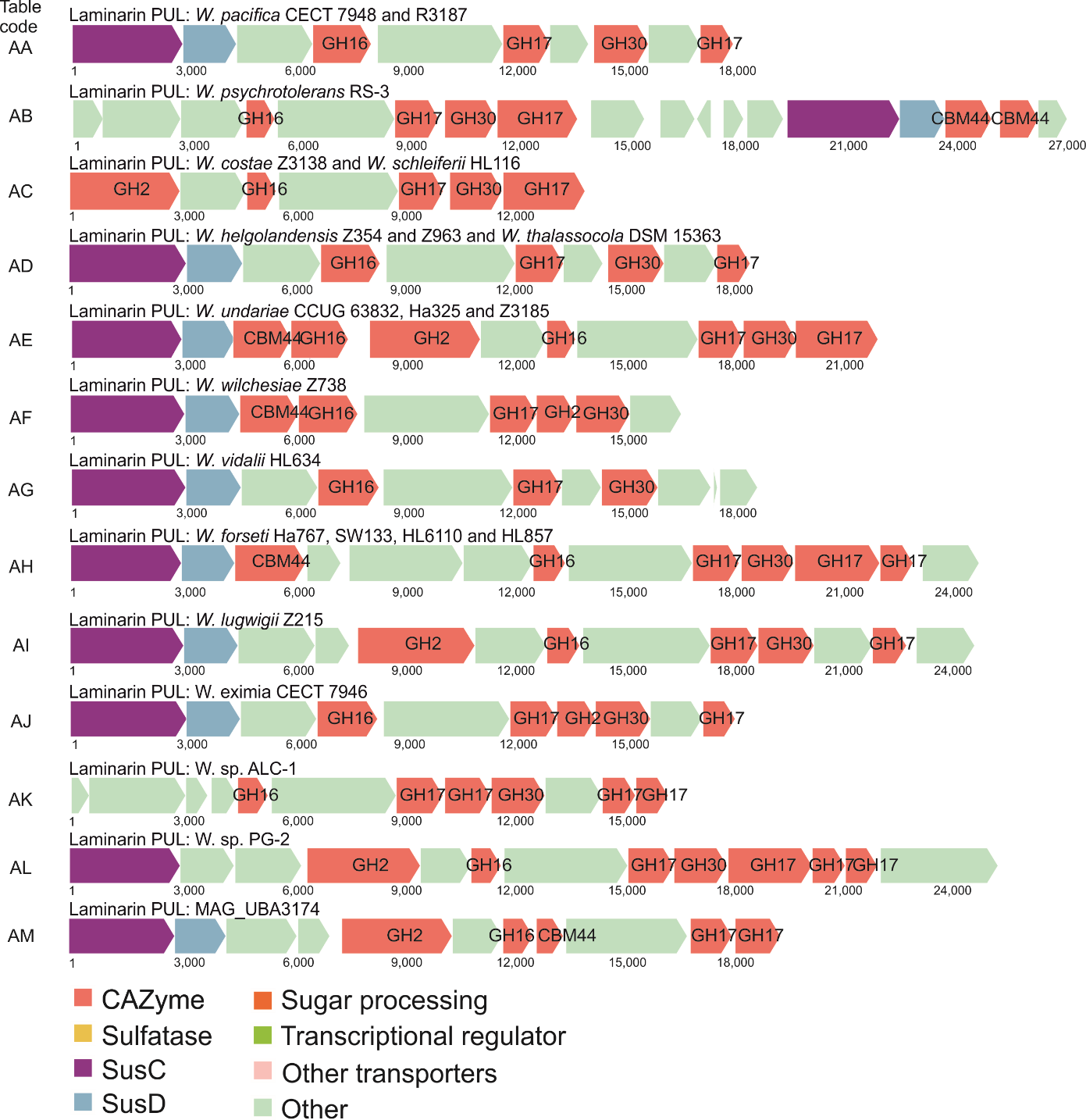
Figure S8B:** Laminarin PULs of the *Winogradskyella* genomes in the study (all located on the larger of the two contigs). Numbers in genes indicate family affiliations of glycoside hydrolases (GH) and carbohydrate-binding modules (CMB).


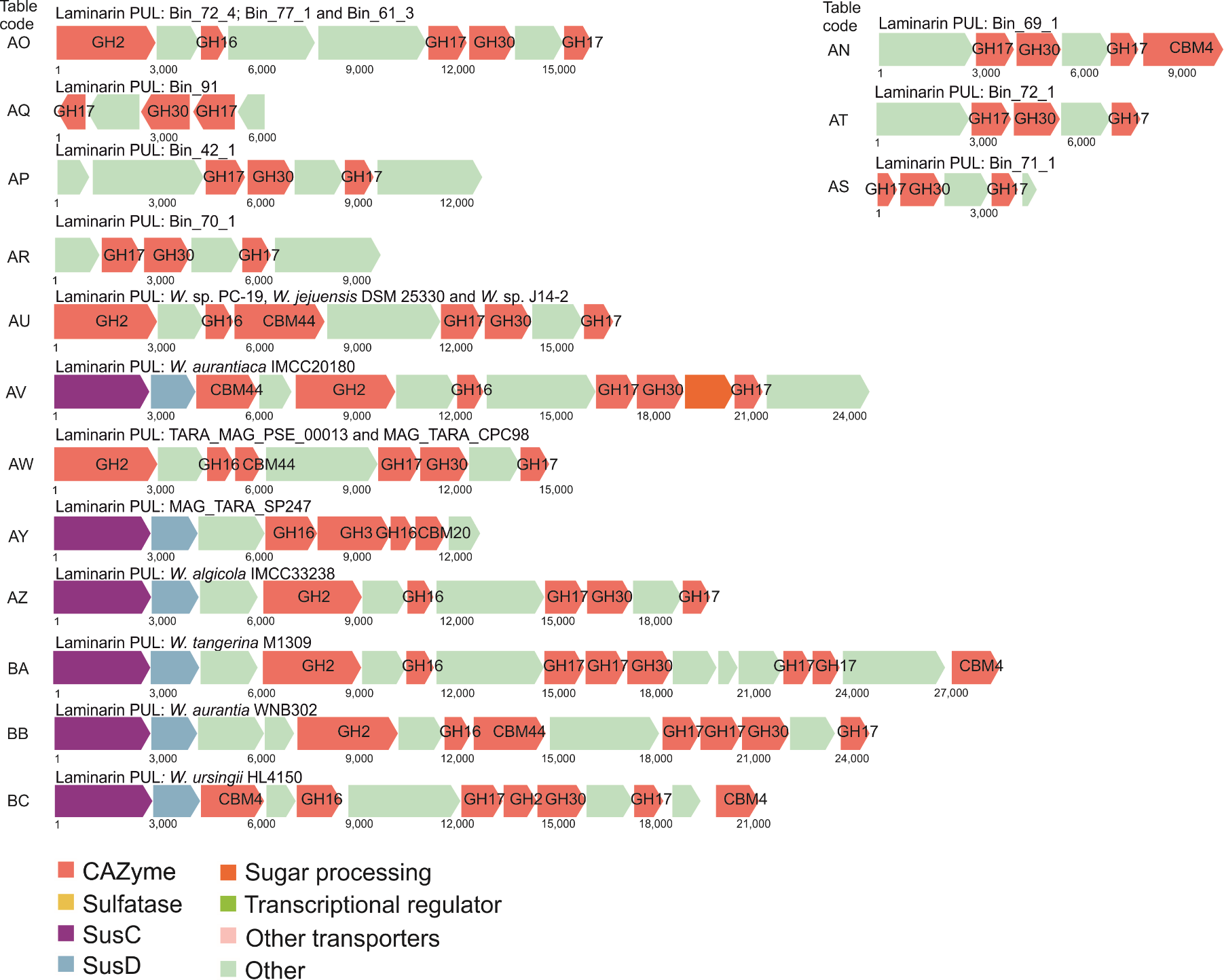


**Supplementary Figure S8C:** Fucoidan PULs of the *Winogradskyella* genomes in the study (all located on the larger of the two contigs). Numbers in genes indicate family affiliations of glycoside hydrolases (GH) and carbohydrate esterases (CE).


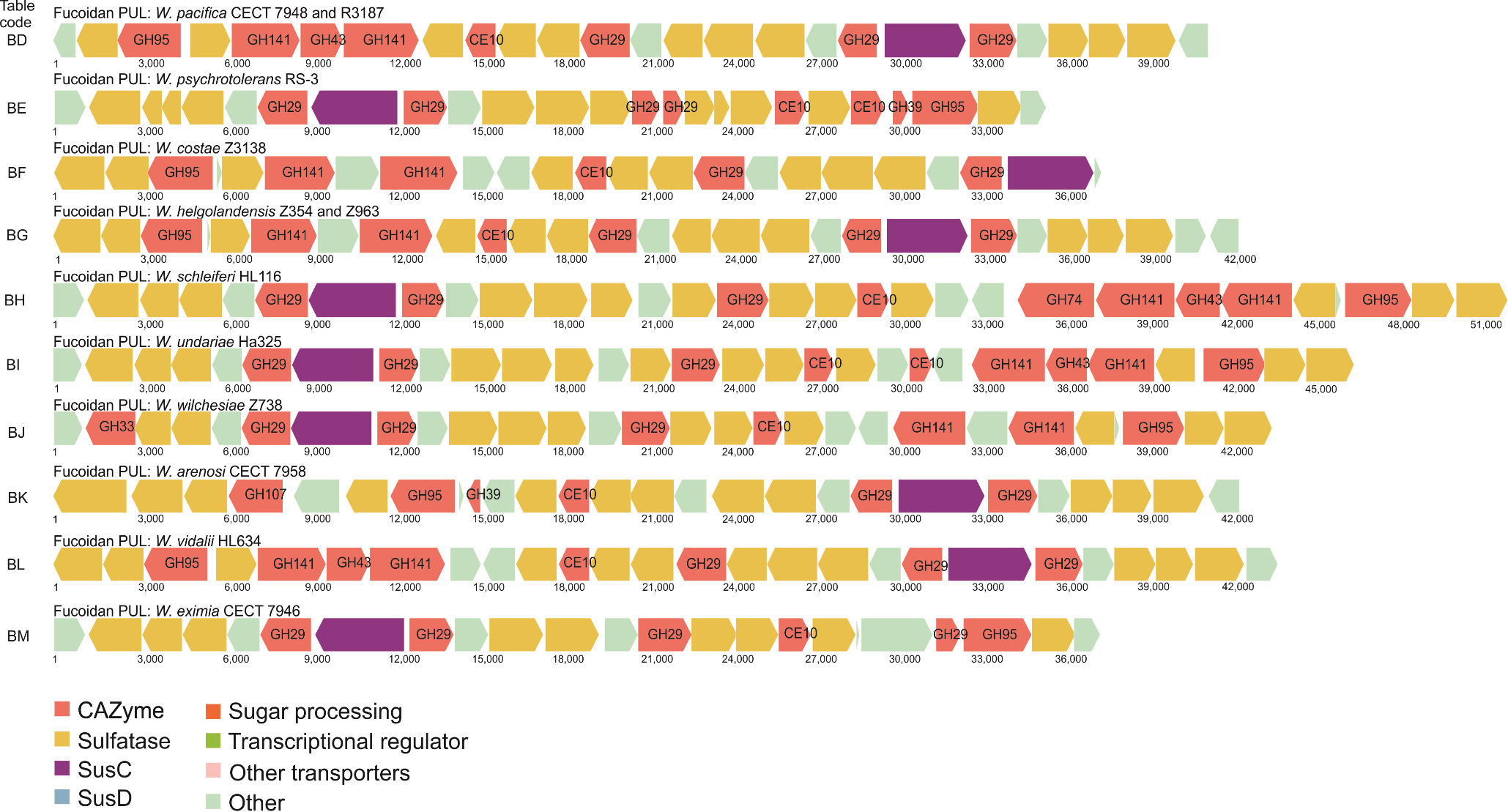


**Supplementary
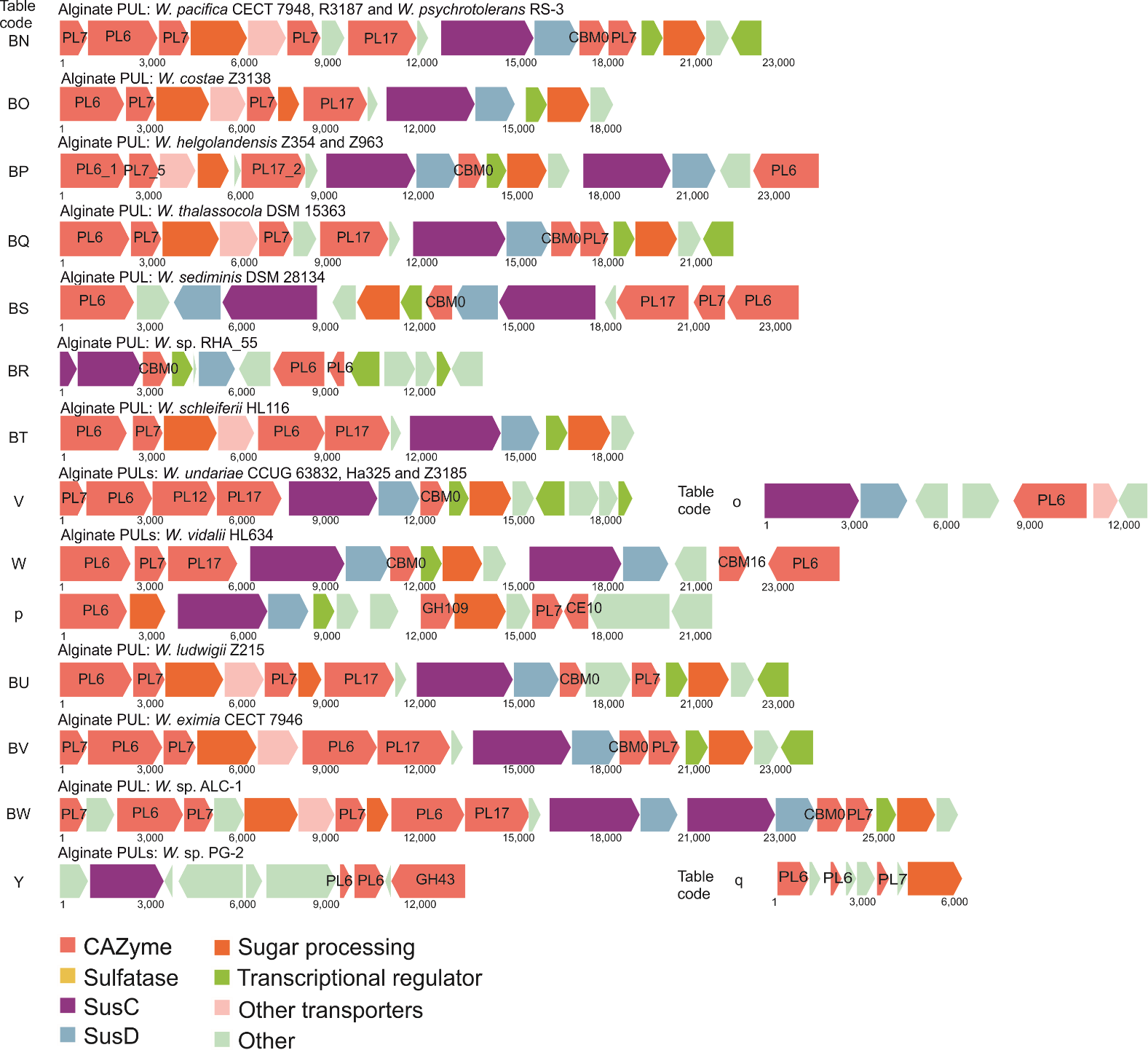
Figure S8D:** Alginate PULs of the *Winogradskyella* genomes in the study (all located on the larger of the two contigs). Numbers in genes indicate family affiliations of polysaccharide lyases (PL), carbohydrate esterases (CE) and carbohydrate-binding modules (CMB).

**Supplementary
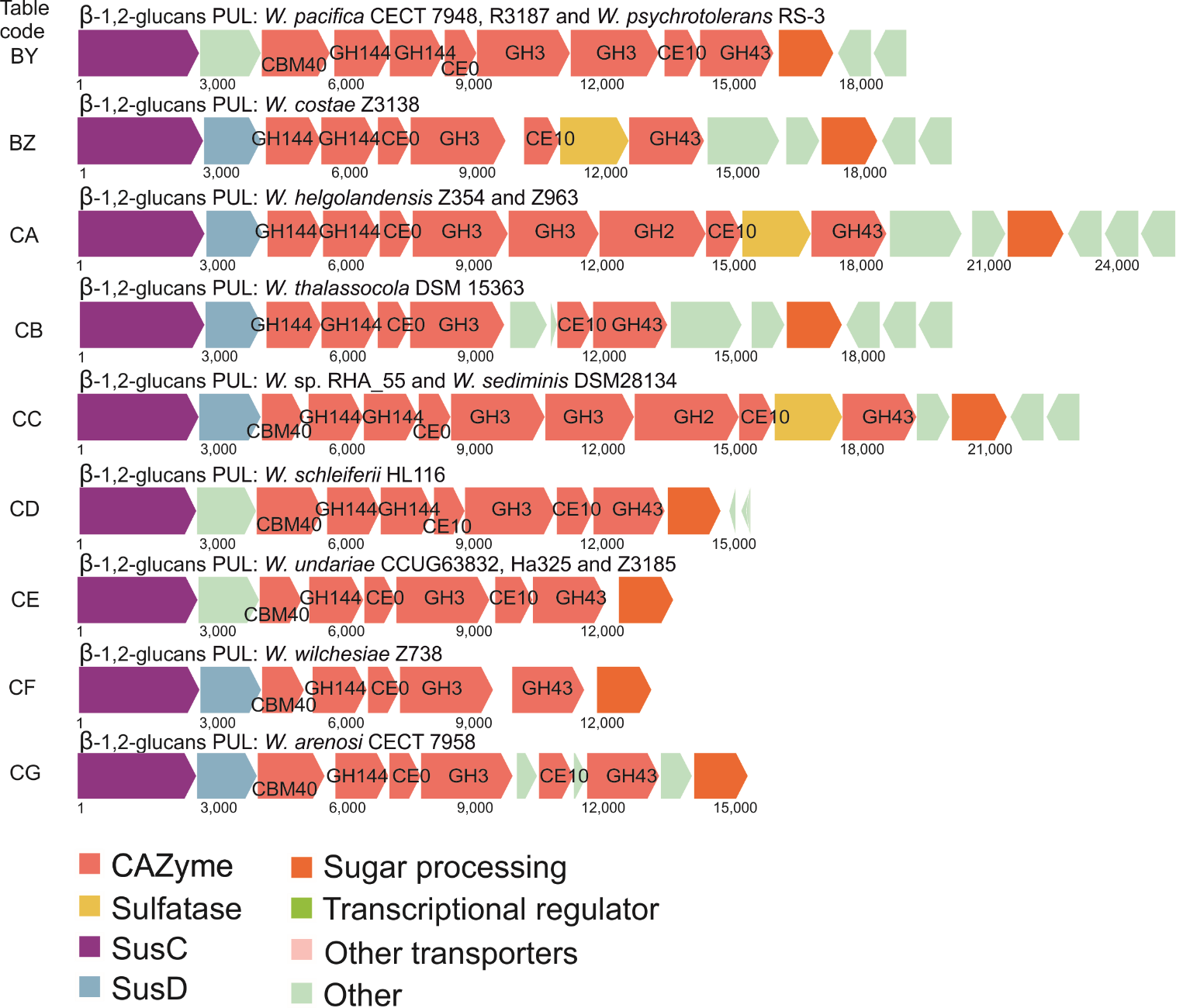
Figure S8E:** β-1,2-glucans PULs of the *Winogradskyella* genomes in the study (all located on the larger of the two contigs). Numbers in genes indicate family affiliations of glycoside hydrolases (GH), carbohydrate esterases (CE) and carbohydrate-binding modules (CMB).


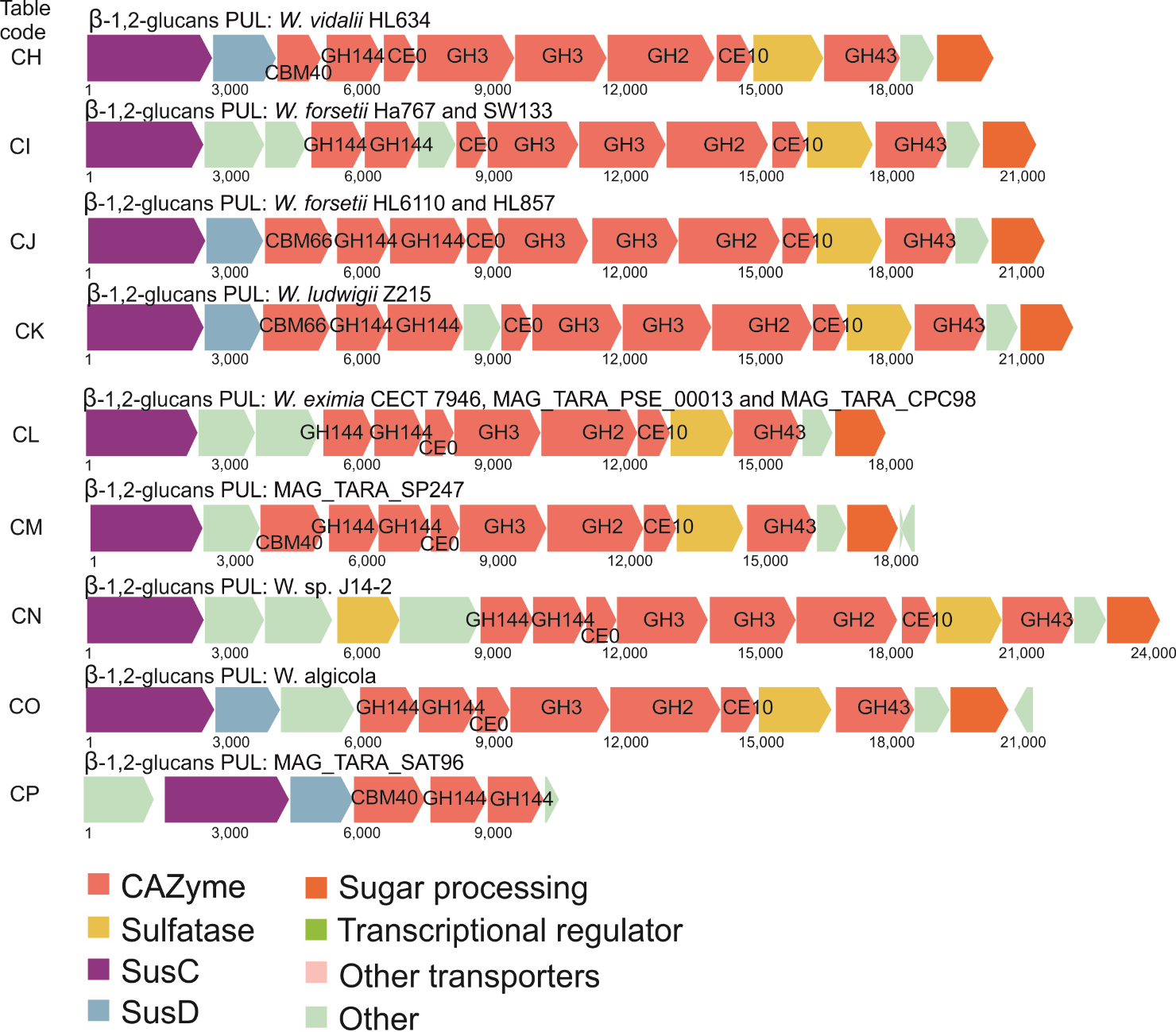


**Supplementary
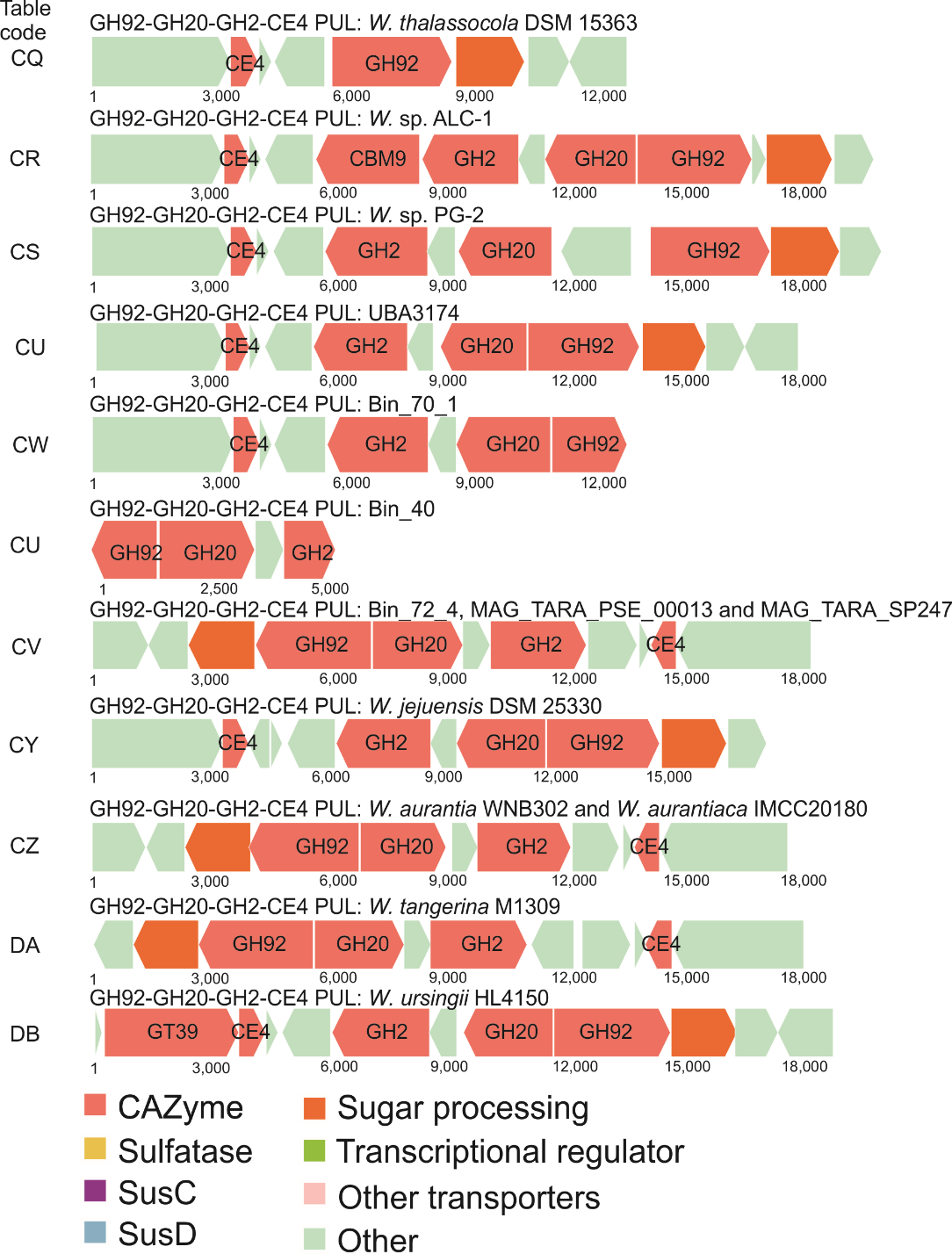
Figure S8F:** GH92-GH20-GH2-CE4 PULs of the *Winogradskyella* genomes in the study (all located on the larger of the two contigs). Numbers in genes indicate family affiliations of glycoside hydrolases (GH), carbohydrate esterases (CE) and glycosyltransferases (GT).

**Supplementary Figure S8G:** Mannan and xylan PULs of the *Winogradskyella* genomes in the study (all located on the larger of the two contigs). Numbers in genes indicate family affiliations of glycoside hydrolases (GH) and carbohydrate esterases (CE).


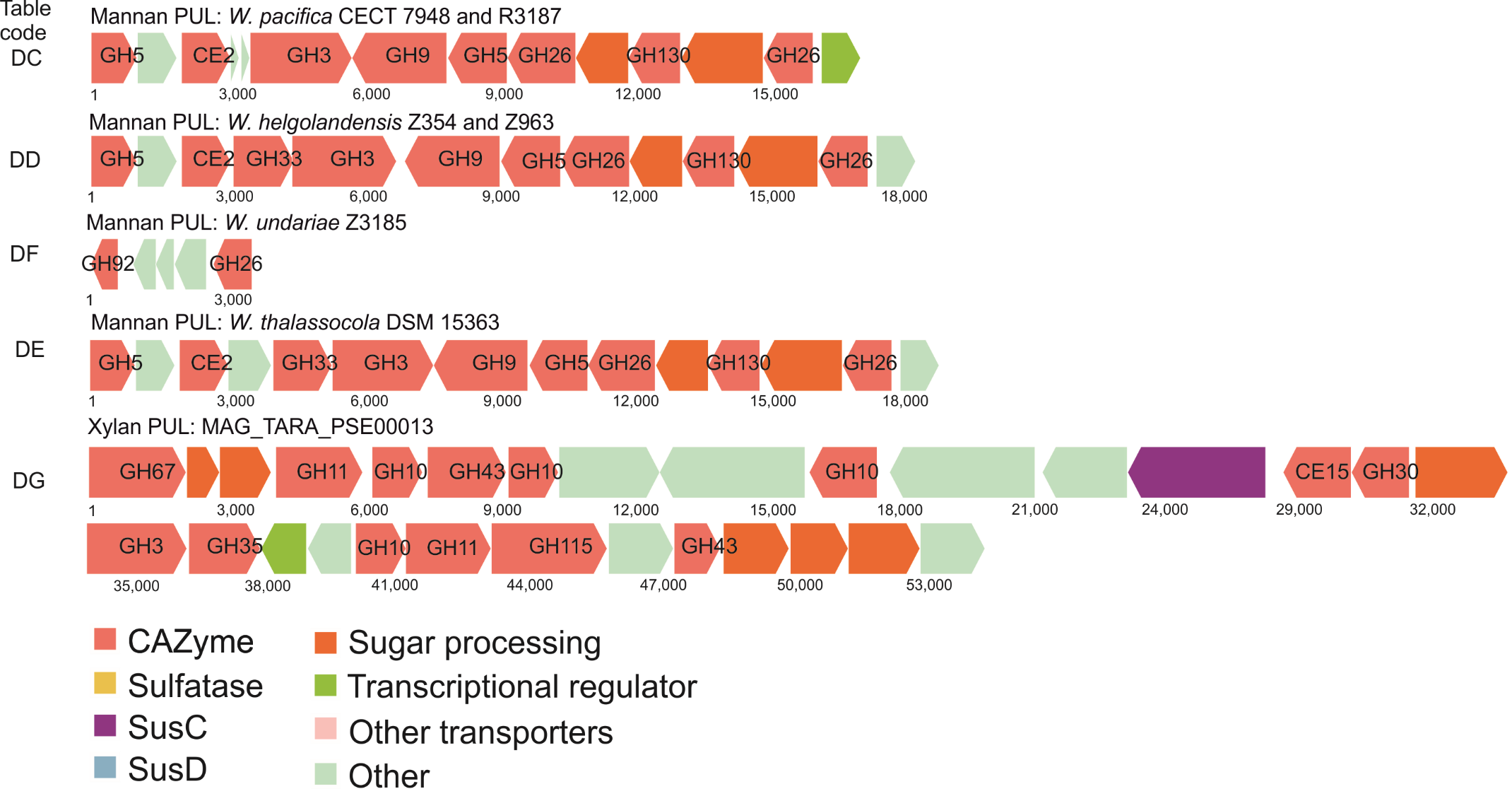


**Supplementary Figure S8H:** Pectin, fucosylated-xylan, sulphated rhamnoglucuran and GH136-sulfated PULs of the *Winogradskyella* genomes in the study (all located on the larger of the two contigs). Numbers in genes indicate family affiliations of glycoside hydrolases (GH), carbohydrate esterases (CE), polysaccharide lyases (PL) and carbohydrate-binding modules (CMB).


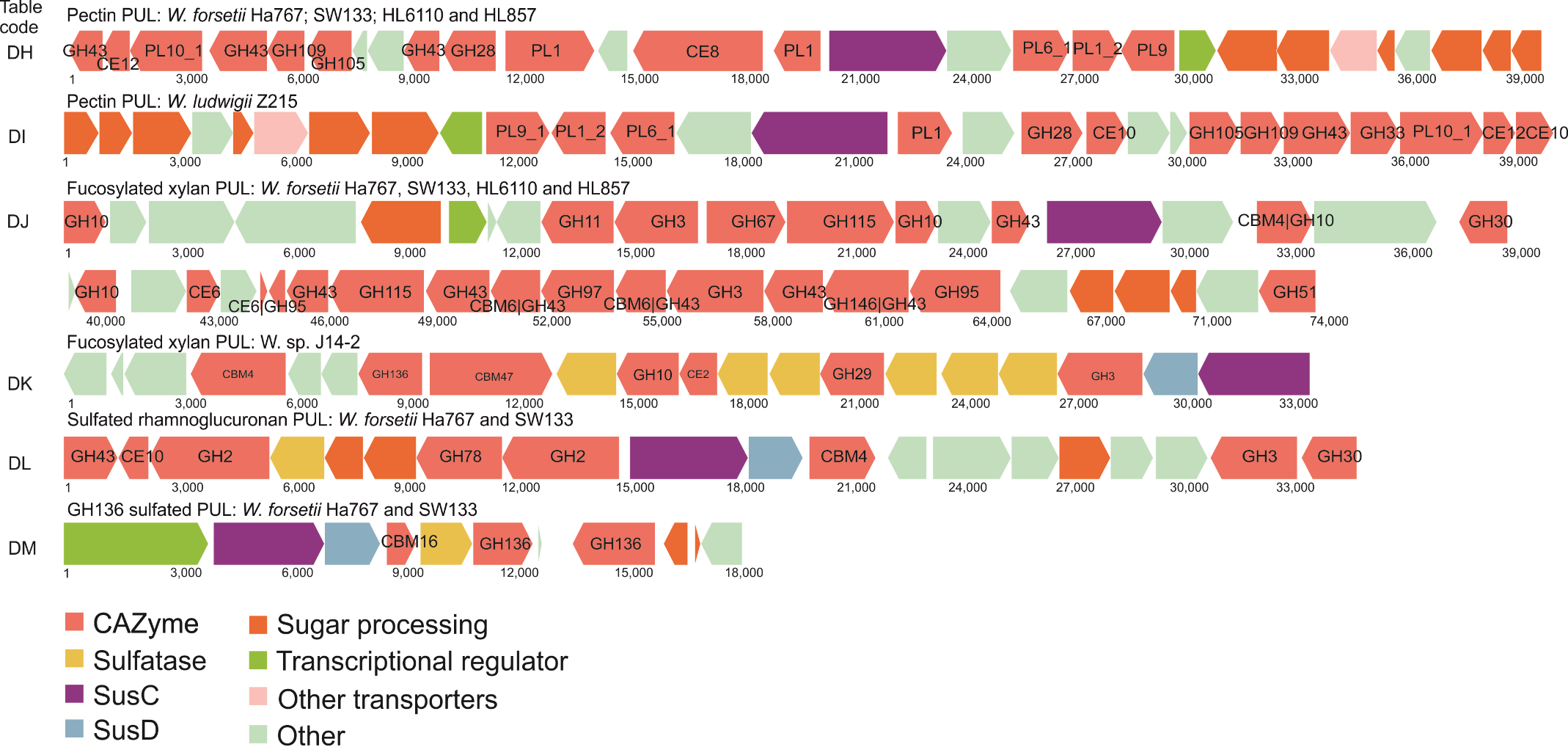


**Supplementary Figure S9:** Recruitment plot example of abundances in metagenomes for Supplementary Table 5.


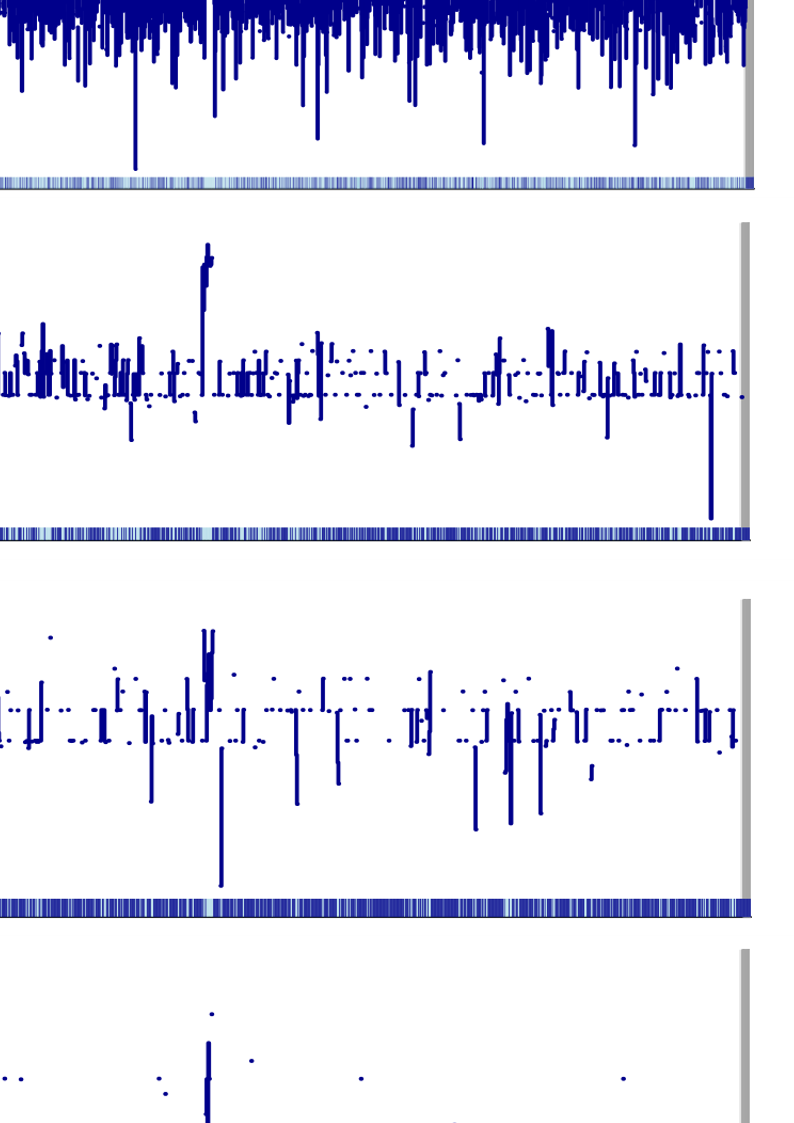


**Detected but no values**

**Detected with values**

**Not detected**
